# Supplementary material for: NMR Experiments Shed New Light on Glycan Recognition by Human and Murine Norovirus Capsid Proteins
Source: Viruses. 2021 Mar 5;13(3):416. doi: 10.3390/v13030416 (PMC8001558; doi:10.3390/v13030416)
Supplement: Supplementary file 1 [file viruses-13-00416-s001.pdf]

Supporting Information

NMR experiments shed new light on glycan recognition by human and murine norovirus capsid proteins

Robert Creutznacher[1], Thorben Maass[1], Patrick Ogrissek[1], Georg Wallmann[1], Clara Feldmann[1], Hannelore Peters[1], Marit Lingemann[2], Stefan Taube[2]*, Thomas Peters[1]*, and Alvaro Mallagaray[1]

[1] University of Lübeck, Institute of Chemistry and Metabolomics, Ratzeburger Allee 160, 23652 Lübeck, Germany; E-mail: thomas.peters@uni-luebeck.de
[2] University of Lübeck, Institute of Virology and Cell Biology, Ratzeburger Allee 160, 23652 Lübeck, Germany; E-mail: stefan.taube@uni-luebeck.de
* Correspondence: thomas.peters@uni-luebeck.de; Tel.: +49 451 3101 3300 (T.P., NMR part); stefan.taube@uni-luebeck.de; Tel.: +49 451 3101 4022 (S.T., Biology part)

Table of Contents

Fig S1. $^{1}H,^{15}N$ TROSY HSQC spectra of [U-$^{2}H,^{15}N$]-labeled P-dimers and sequence alignment of GII.4 strains. 3
Fig S2. Deamidation of GII.4 strains MI001 and Saga. 4
Fig S3. IEX experiments with GII.4 Saga N373D show stability at 25 °C for weeks...5
Fig S4. Re-evaluation of previously reported binding isotherms STD. 6
Fig S5. Binding of Mn^{2+} impurities in chemo-enzymatically synthesized B-trisaccharide to [U-$^{2}H,^{15}N$]-labeled MNV CW1 P-domain. 7
Fig S6. Additional CSP titration curves..8
Fig S7: $^{1}H,^{15}N$ TROSY HSQC spectra showing no binding of sialylated ganglioside glycans to human NoV GII.4 P-domains as summarized in Table 3. 9
Fig S8: $^{1}H,^{15}N$ TROSY HSQC and STD NMR spectra showing no binding of sialylated ganglioside glycans to murine NoV P-domains CW1 and MNV07 as summarized in Table 2...19
Table S1. Experimental conditions for data in Table 2. 42
Table S2. Source of glycans. 43
Table S3: Assignment of blood group B trisaccharide 3...44
Table S4: NMR experiments and experimental conditions used for the assignment of blood group B trisaccharide 3. 45
Table S5. Reevaluation of dissociation constants K_D based on previously reported titrations..............................46
References:...46

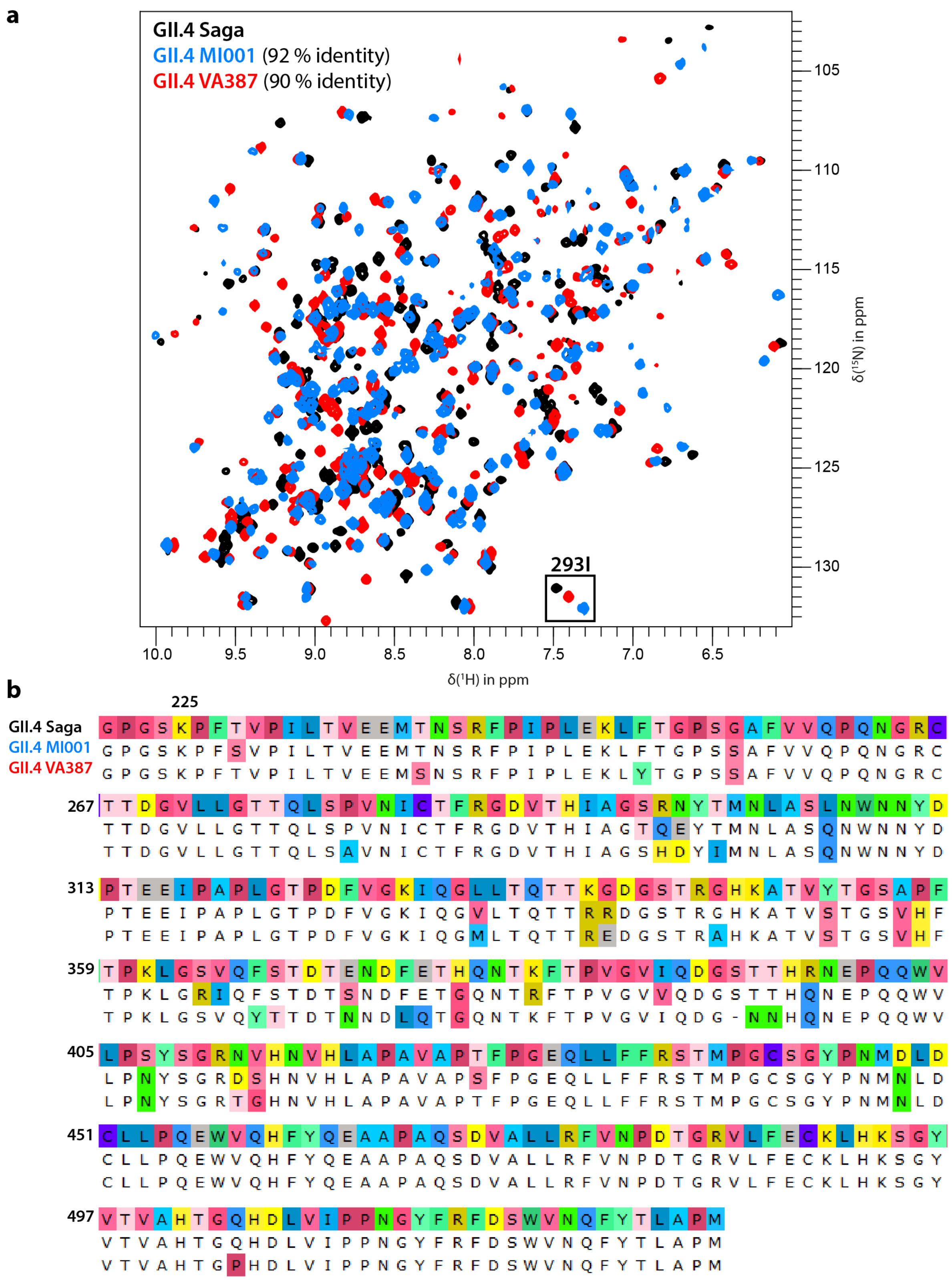

Fig S1. ^{1}H,^{15}N TROSY HSQC spectra of [*U*-^{2}H,^{15}N]-labeled P-dimers and sequence alignment of GII.4 strains.

a) ^{1}H,^{15}N TROSY HSQC spectra of [*U*-^{2}H,^{15}N]-labeled P-dimers **of** different GII.4 strains (Saga: black, MI001: blue, VA387: red). Signal assignments can be transferred from the assigned GII.4 Saga spectrum to spectra of the homologous P-domains in isolated spectral regions. An example is highlighted with a black box. b) Sequence alignment of the respective GII.4 P-domains (generated with UGENE **[1]**).

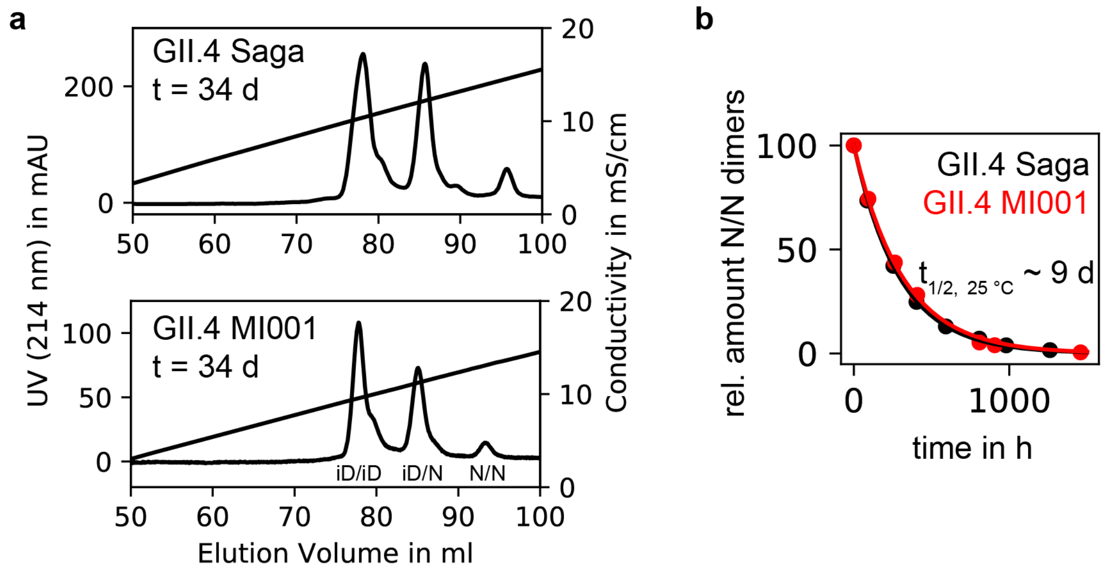

Fig S2. Deamidation of GII.4 strains MI001 and Saga.
Exemplary IEX chromatograms shown in a after incubation of protein aliquots in 75 mM sodium phosphate, 100 mM NaCl (pH 7.3) at 25 °C. Decay curve of the N/N species (b) was obtained by integration of the respective peaks in UV chromatograms at 214 nm acquired at selected time points. Data were fitted to a simple exponential decay model. The half-lives of both proteins are almost identical.

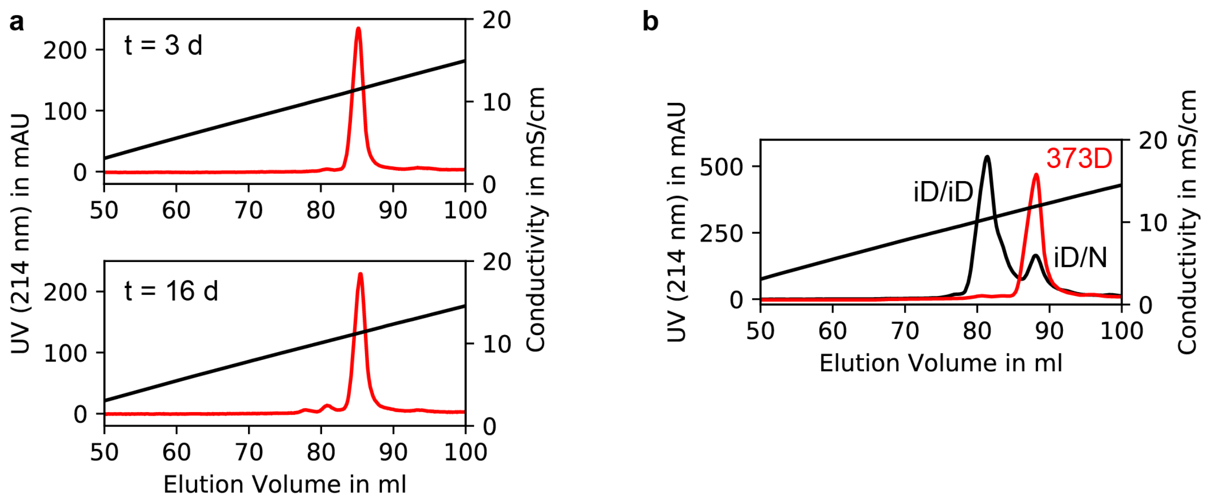

Fig S3. IEX experiments with GII.4 Saga N373D show stability at 25 °C for weeks.
Prolonged incubation of protein aliquots in 75 mM sodium phosphate, 100 mM NaCl (pH 7.3) at 25 °C shows no substantial interconversion of aspartate into iso-aspartate as evidenced by unchanged IEX chromatograms. IEX analysis can discriminate between isoAsp and Asp as both have different pKa values leading to a sufficient charge difference at the pH of the running buffer (4.9). An overlay of IEX chromatograms of an aged, mostly deamidated sample of GII.4 Saga wild-type and the N373D point mutant is shown in b for reference.

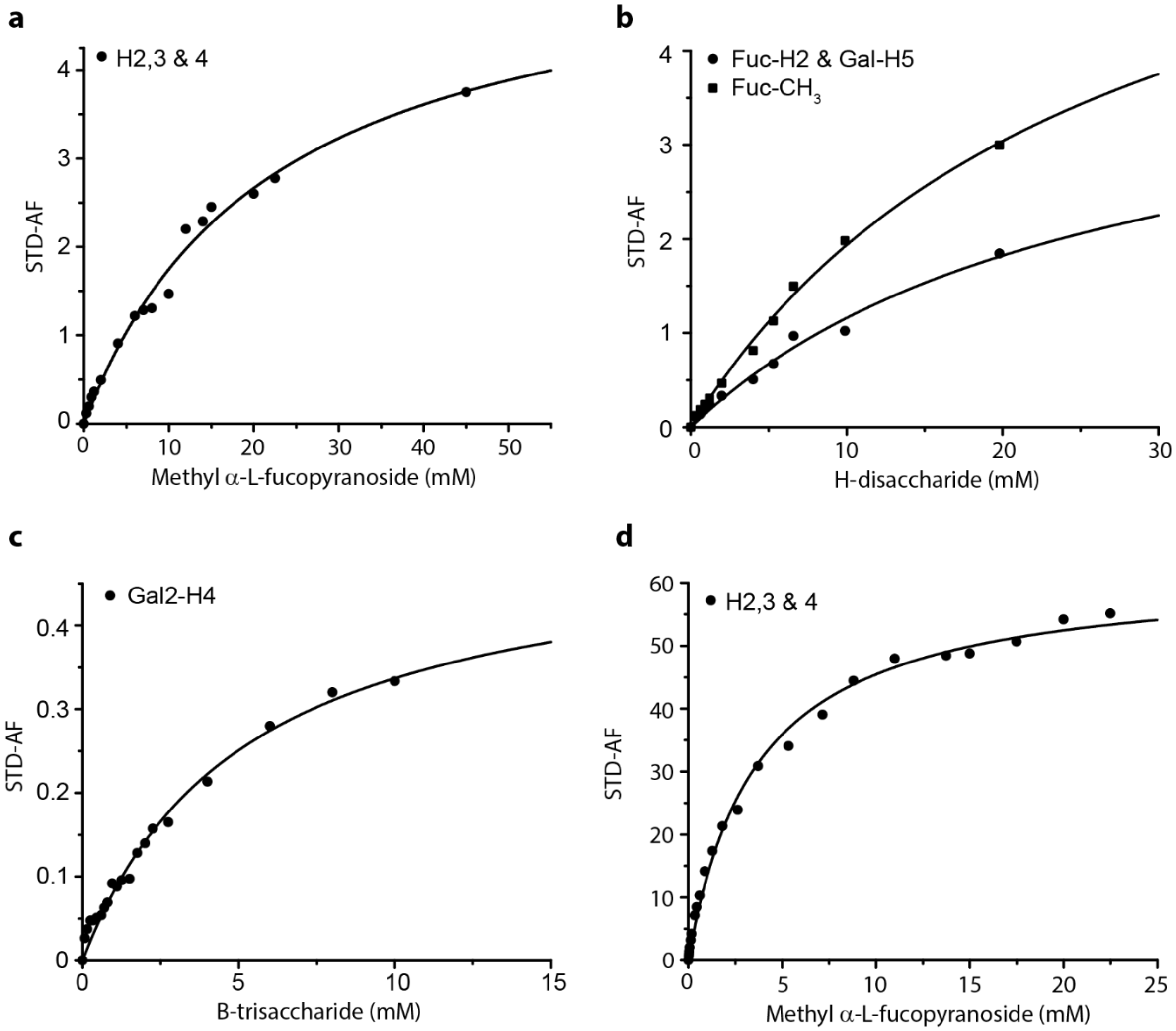

Fig S4. Re-evaluation of previously reported binding isotherms STD.

A one-site binding model was fitted to the data. Binding isotherms correspond to the titration of: a) GII.4 Saga P-domains with methyl α-L-fucopyranoside 8 **[2]**, b) GII.4 Saga P-domains with H-disaccharide 12 **[2]**, c) GII.4 Saga P-domains with the methyl glycoside of B-trisaccharide **[2]**, d) GII.4 Ast6139 VLPs with methyl α-L-fucopyranoside 8 **[3]**. Dissociation constants K_D are listed in Table S3 (entries 3, 7, 8 and 9, respectively). Detailed experimental conditions can be found in the corresponding publications.

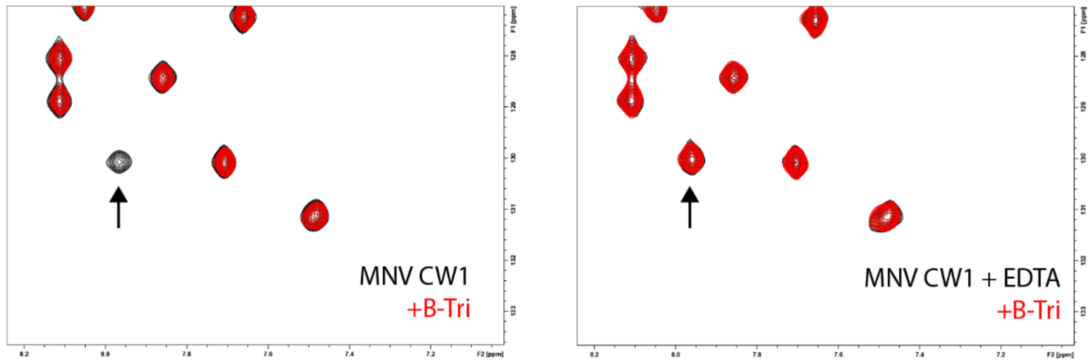

Fig S5. Binding of Mn^{2+} impurities in chemo-enzymatically synthesized B-trisaccharide to [*U*-^{2}H,^{15}N]-labeled MNV CW1 P-domain.

A representative section of ^{1}H,^{15}N TROSY HSQC spectra shows a signal broadened beyond detection upon B-trisaccharide addition (left, indicated by the arrow). The presence of EDTA in the sample prevents this effect (right), indicating that broadening occurs due to paramagnetic relaxation enhancement from a Mn^{2+} contamination. The sample corresponding to the left spectrum contained 200 µM CW1, 500 µM DSS, 600 µM GCDCA in 20 mM NaAc, 100 mM NaCl (pH 5.3) and 4 mM B-trisaccharide. The sample corresponding to the right spectrum contained 3 mM B-trisaccharide and additionally 4 mM EDTA. All spectra were acquired at 500 MHz.

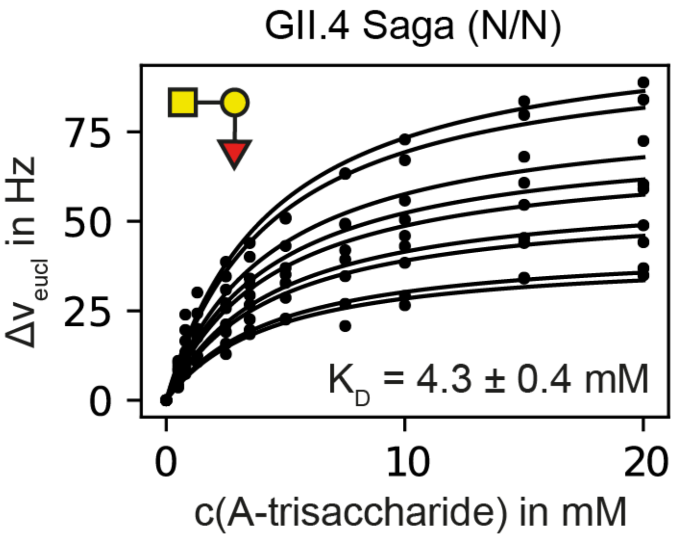

Fig S6. Additional CSP titration curves.
Titration curves for A-Tri 2 binding to [U-^{2}H,^{15}N]-labeled Saga GII.4 P-dimers (cf. Fig. 2 and Table 1 of the main text).

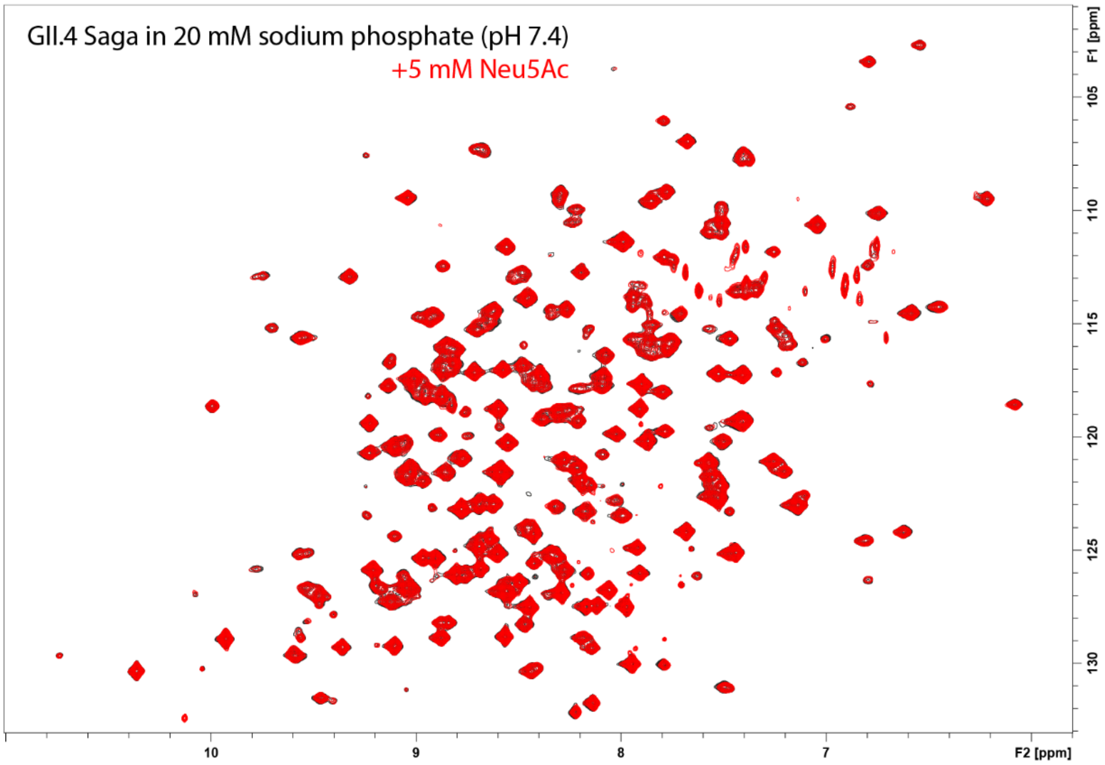

Fig S7: ^{1}H,^{15}N TROSY HSQC spectra showing no binding of sialylated ganglioside glycans to human NoV GII.4 P-domains as summarized in Table 3.

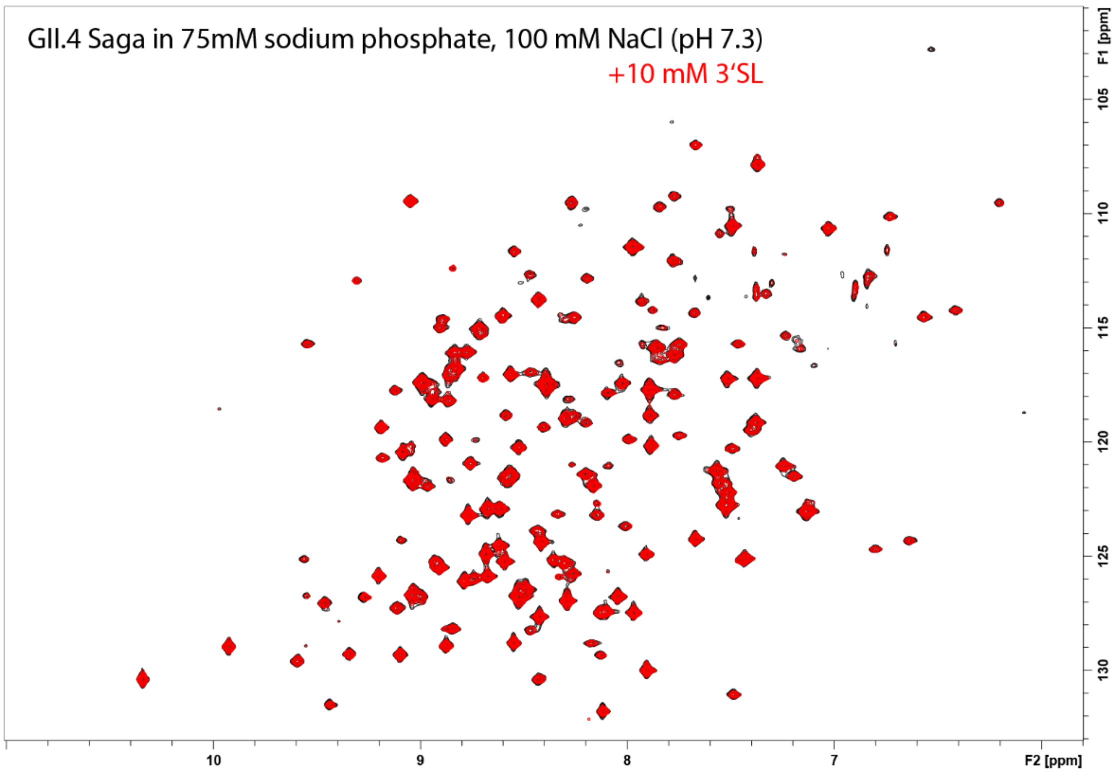

Fig S7 [continued]

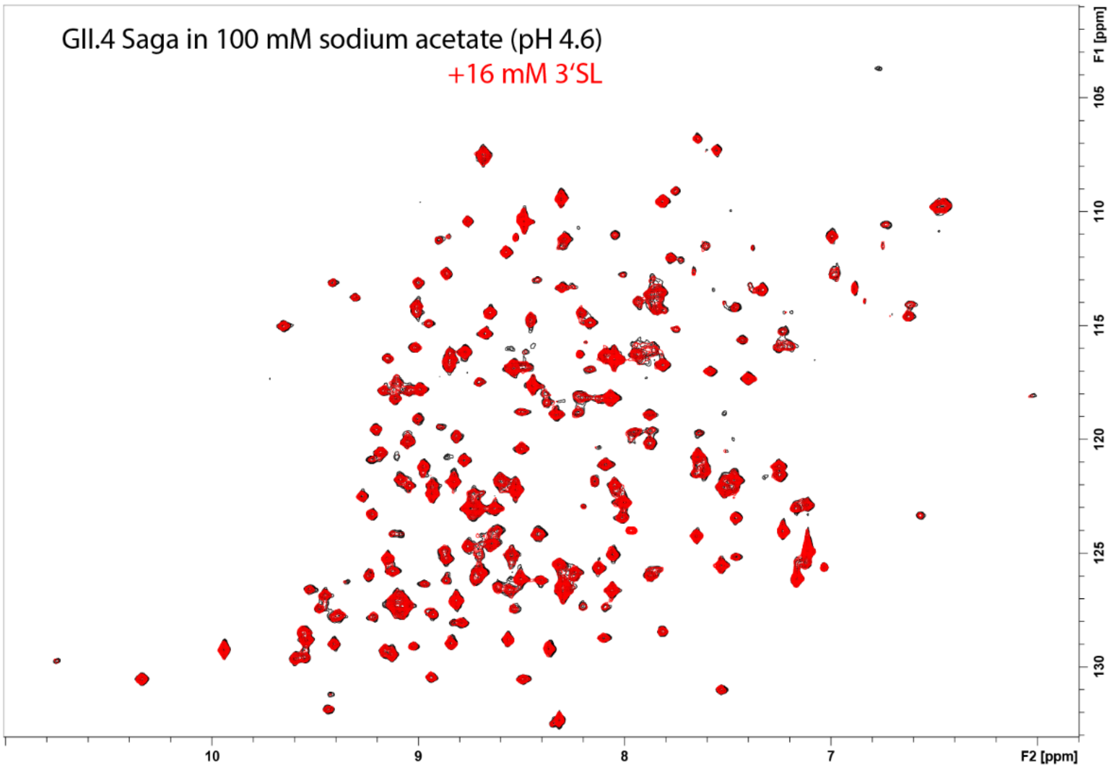

Fig S7 [continued]

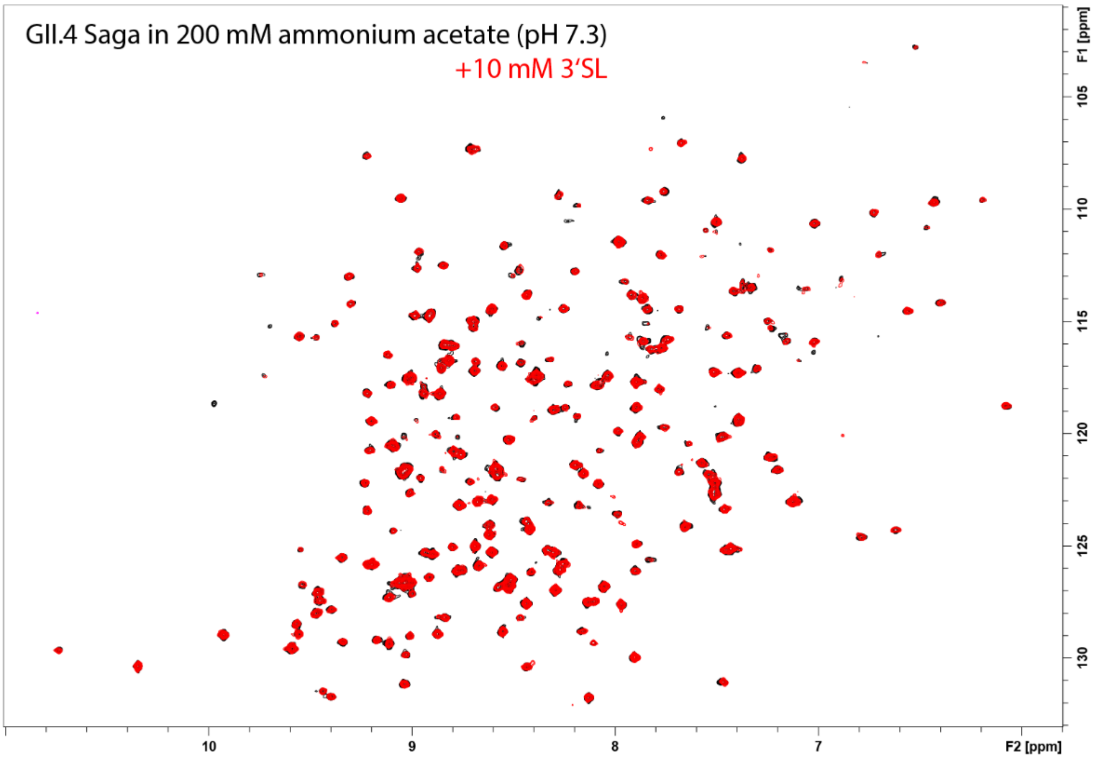

Fig S7 [continued]

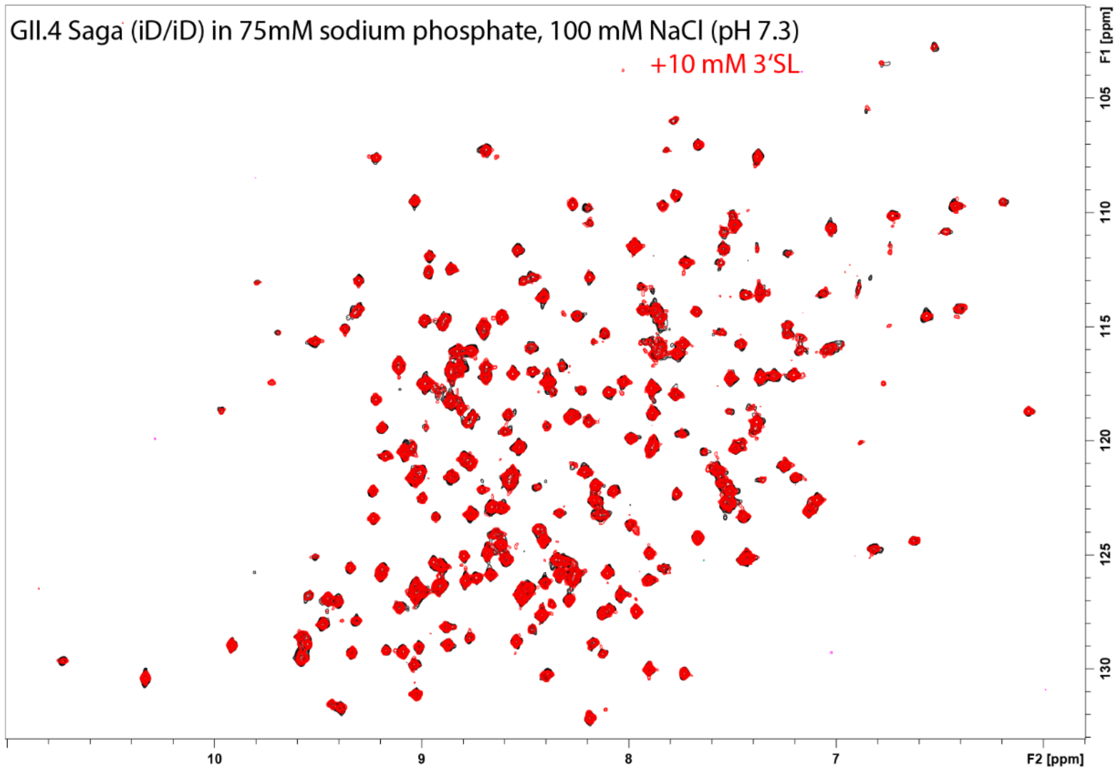

Fig S7 [continued]

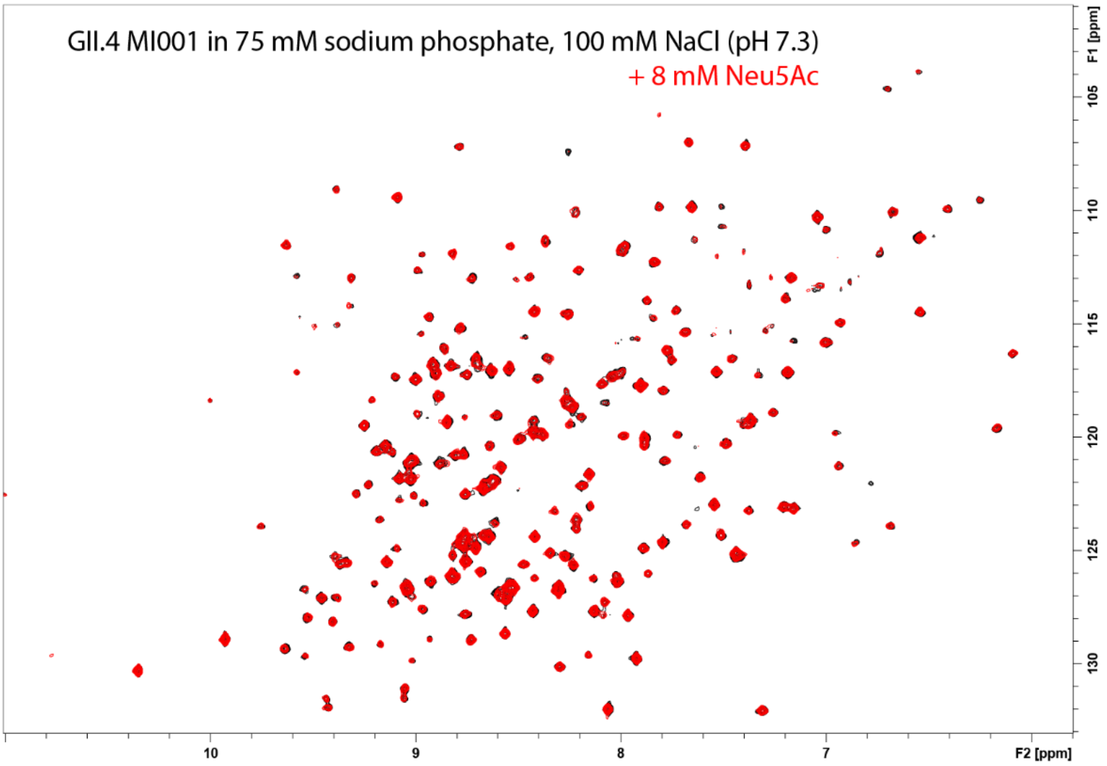

Fig S7 [continued]

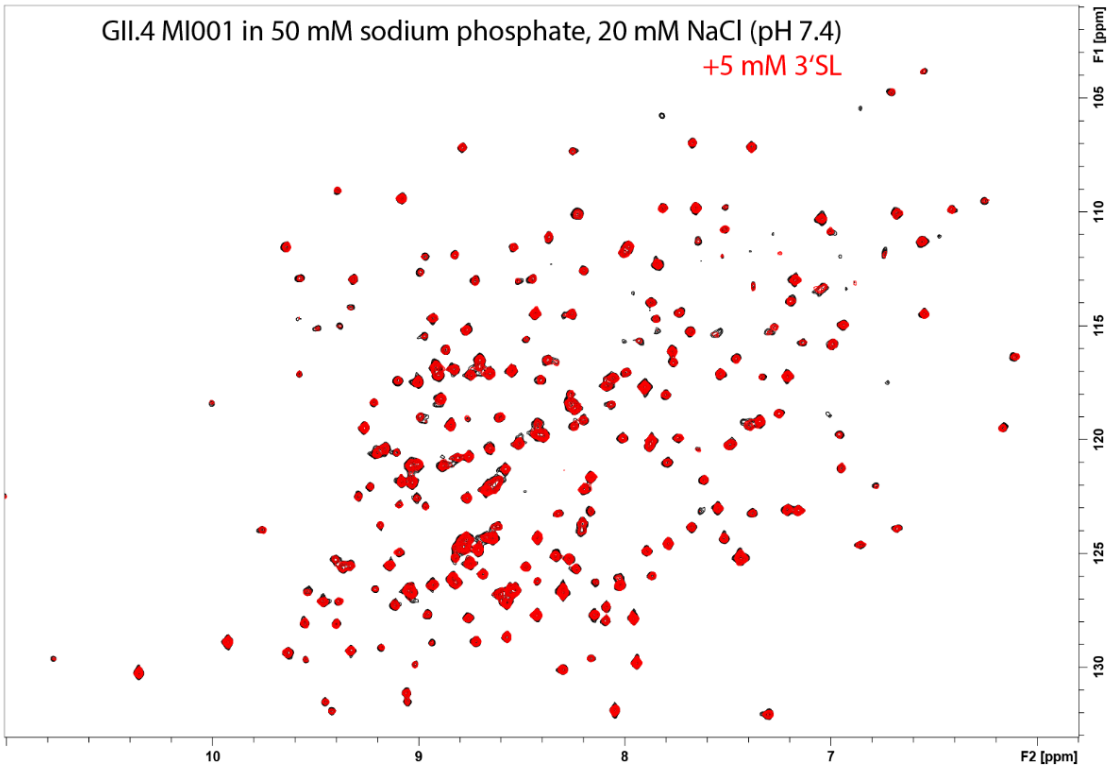

Fig S7 [continued]

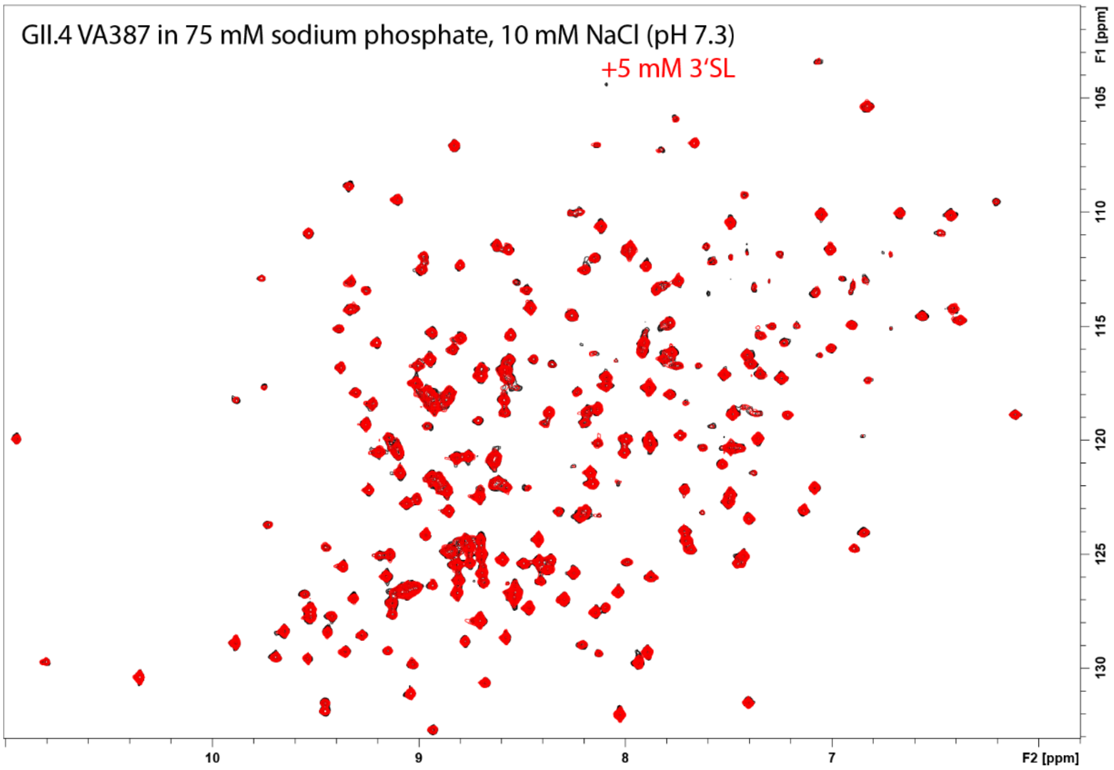

Fig S7 [continued]

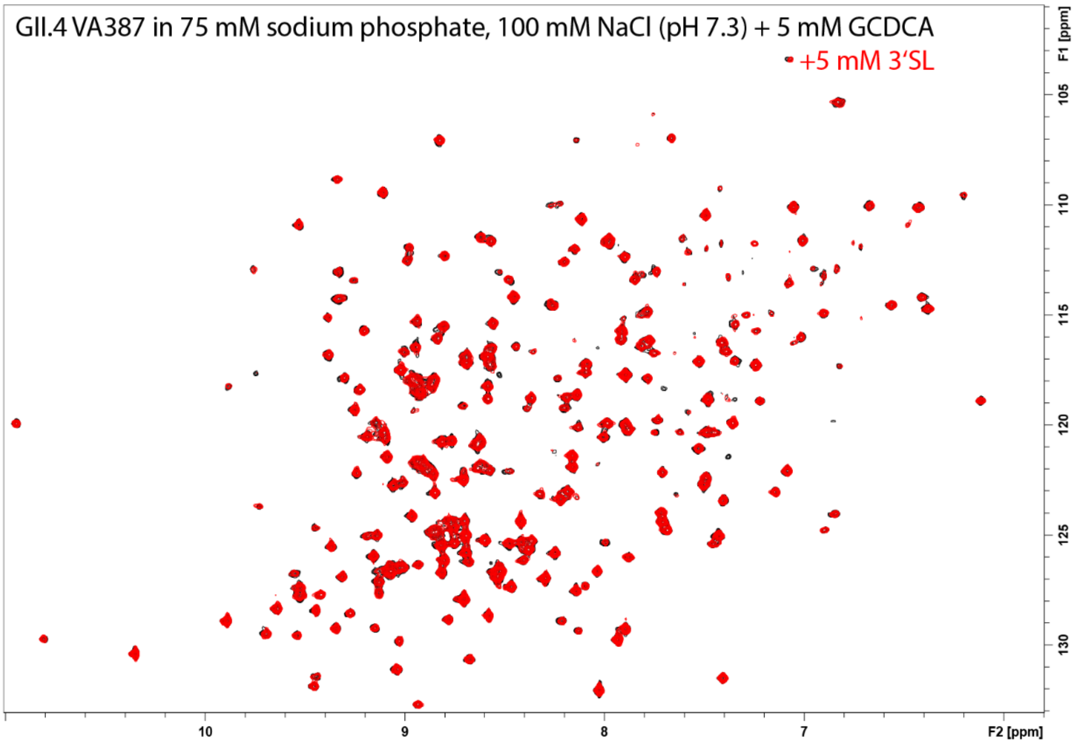

Fig S7 [continued]

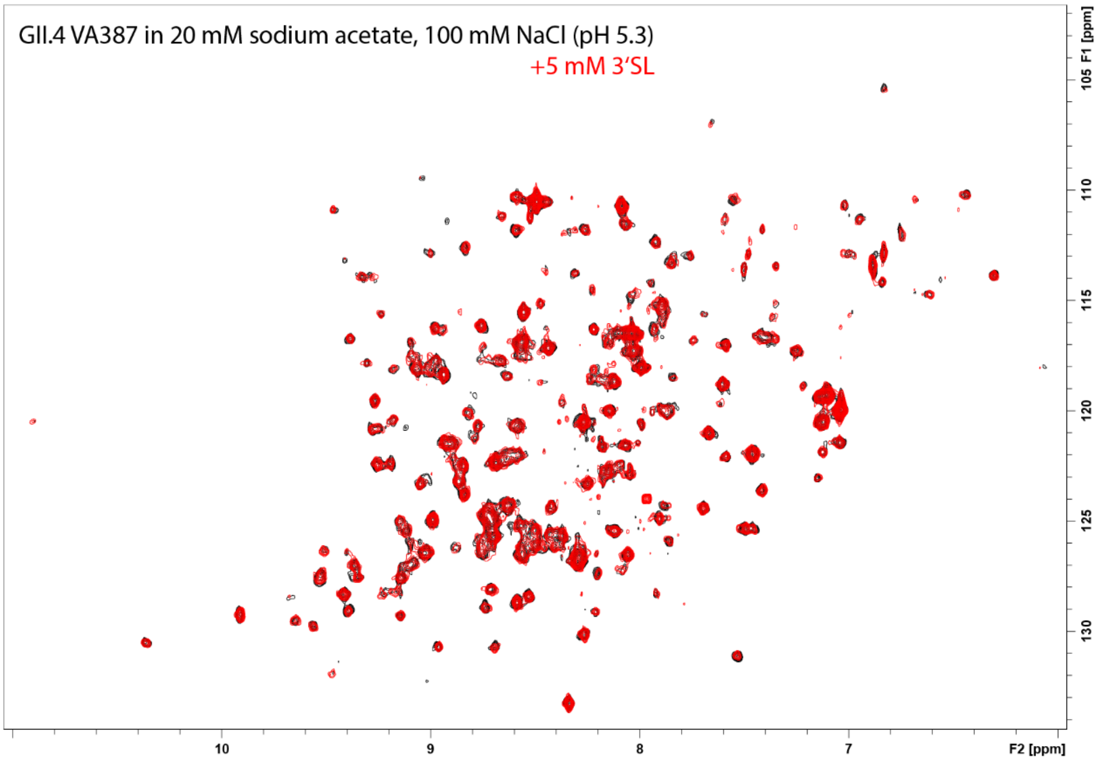

Fig S7 [continued]

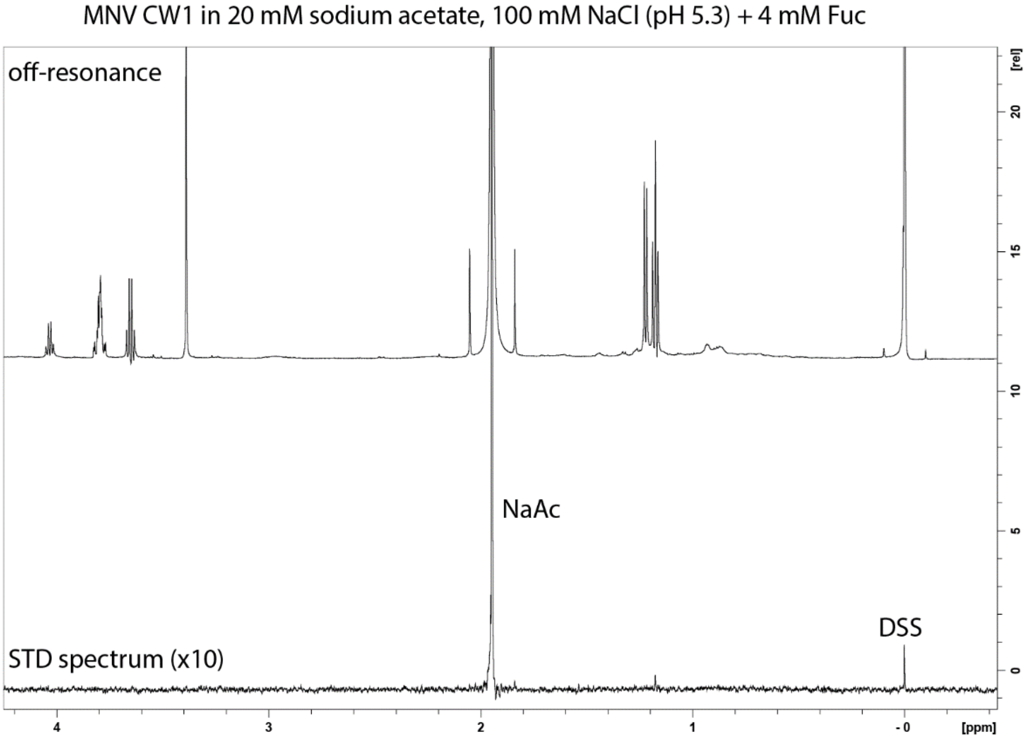

Fig S8: $^{1}H,^{15}N$ TROSY HSQC and STD NMR spectra showing no binding of sialylated ganglioside glycans to murine NoV P-domains CW1 and MNV07 as summarized in Table 2.
Some glycans were found to contain significant amounts of divalent metal ions, e.g. Ca^{2+}, Mn^{2+}, or Mg^{2+}. In these cases, metal binding can cause fast exchange CSPs (cf. Fig 4) or paramagnetic relaxation enhancement respectively. Reference spectra of dedicated metal ion titrations are shown for comparison, arrows denote some characteristic CSPs.

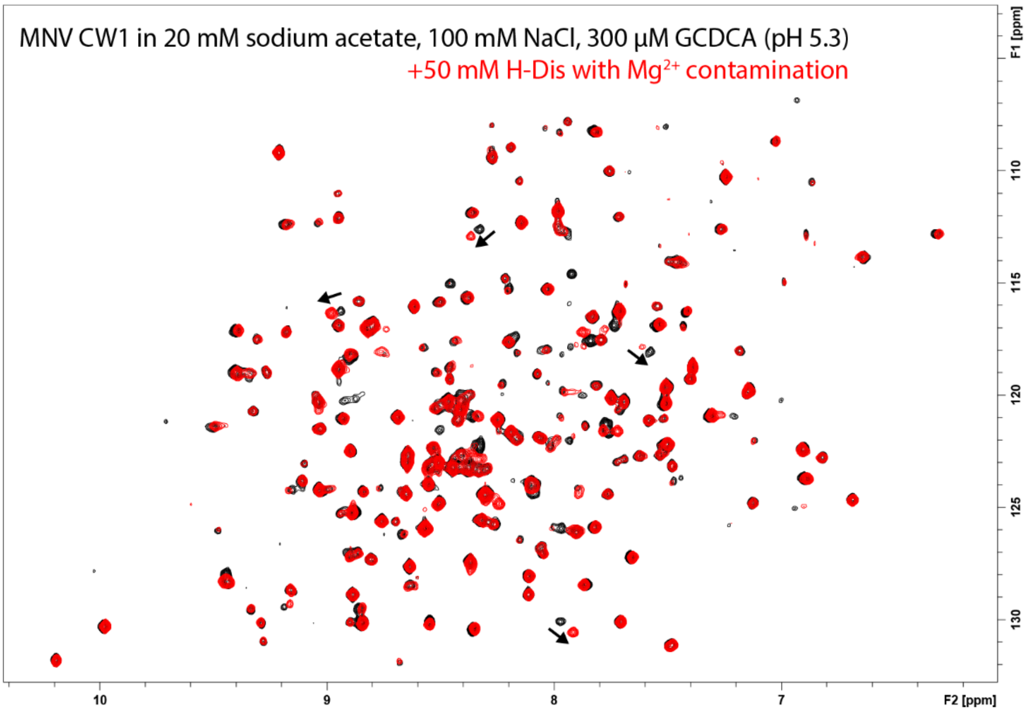

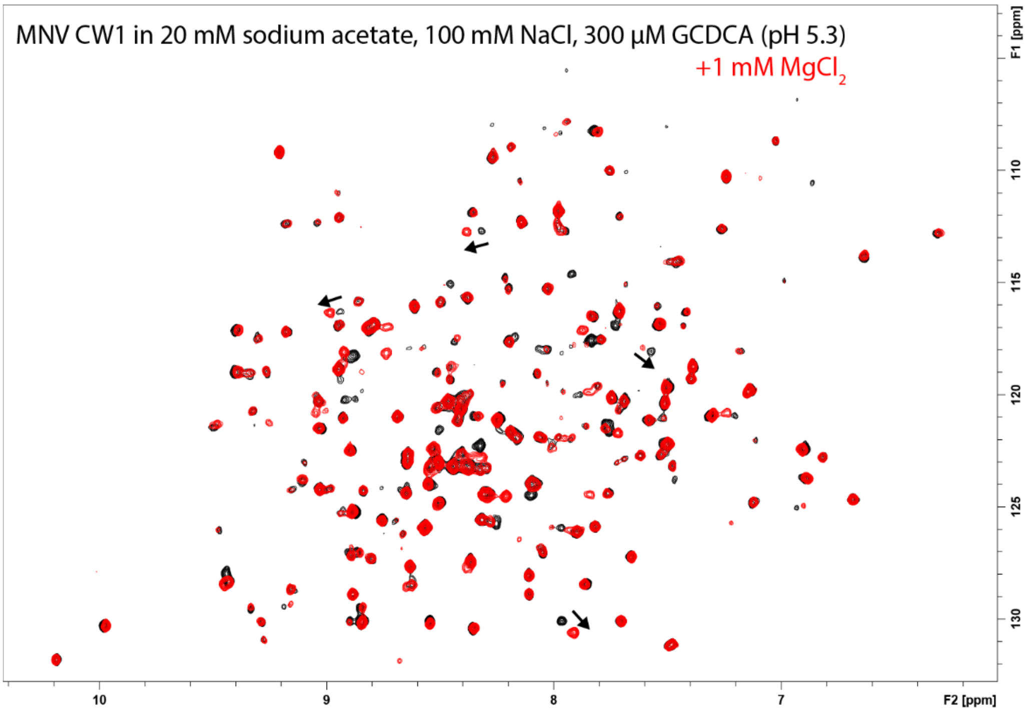

Fig S8 [continued]

MNV CW1 in 20 mM sodium acetate-d_3, 100 mM NaCl (pH 5.3), D_2O, 300 µM GCDCA + 2 mM A-Tri

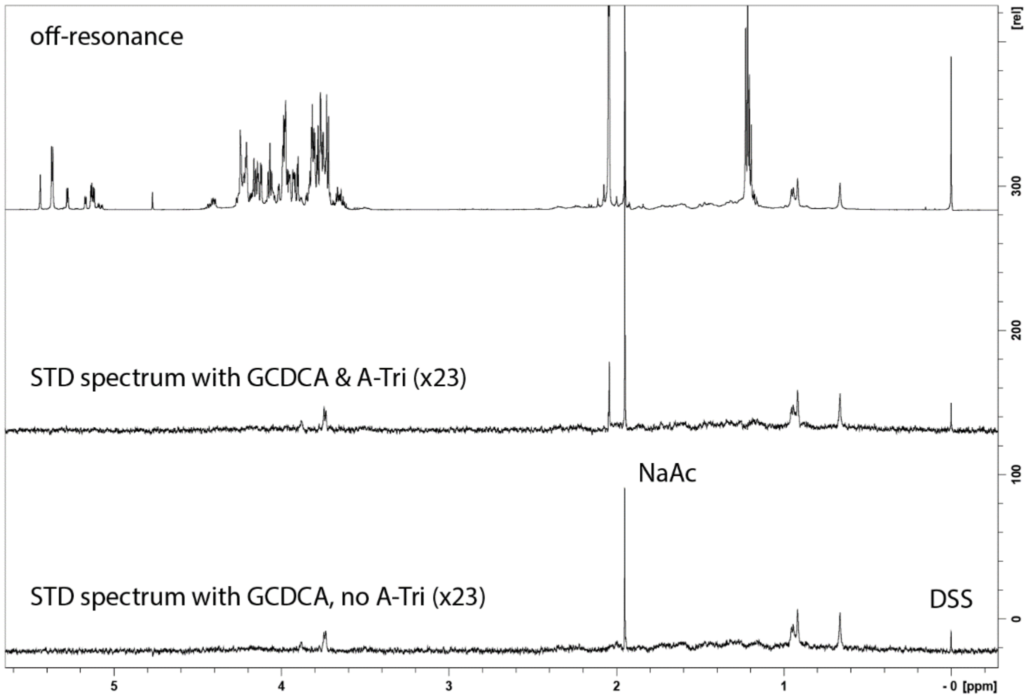

Fig S8 [continued]

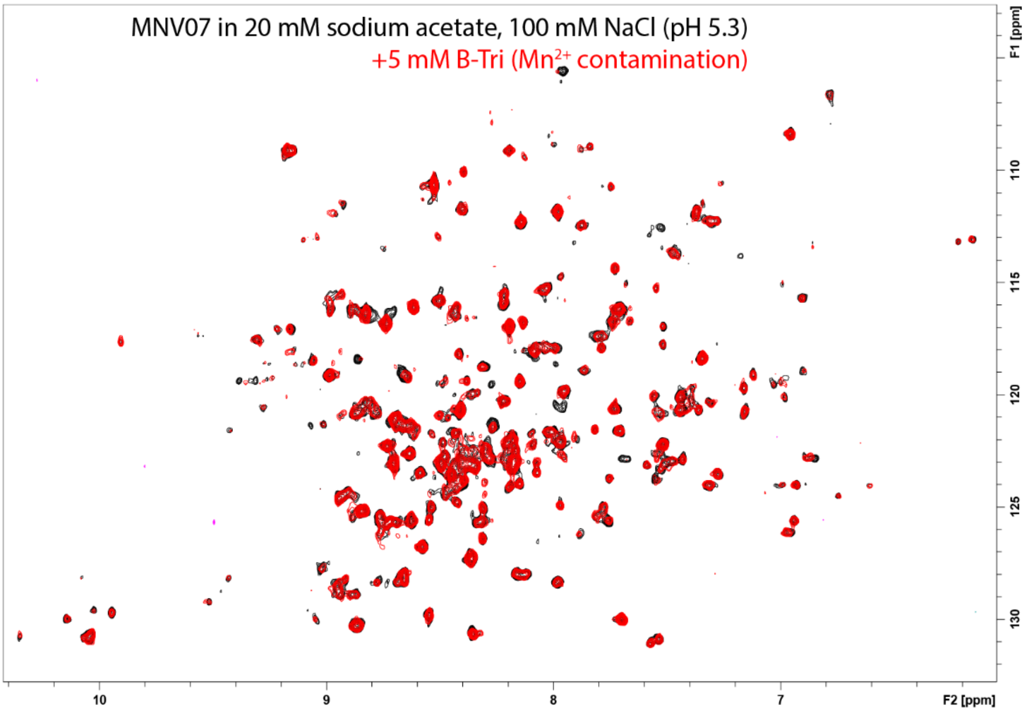

Fig S8 [continued]

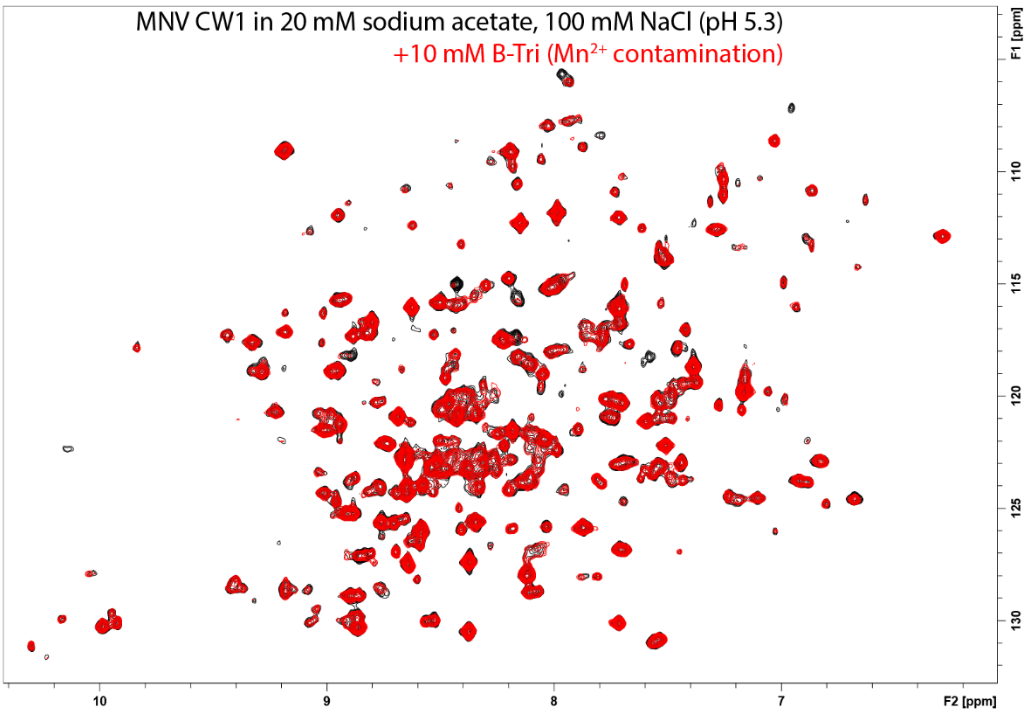

Fig S8 [continued]

MNV07 in 20 mM sodium acetate, 100 mM NaCl (pH 5.3),140 µM GCDCA + 1.8 mM B-Tri

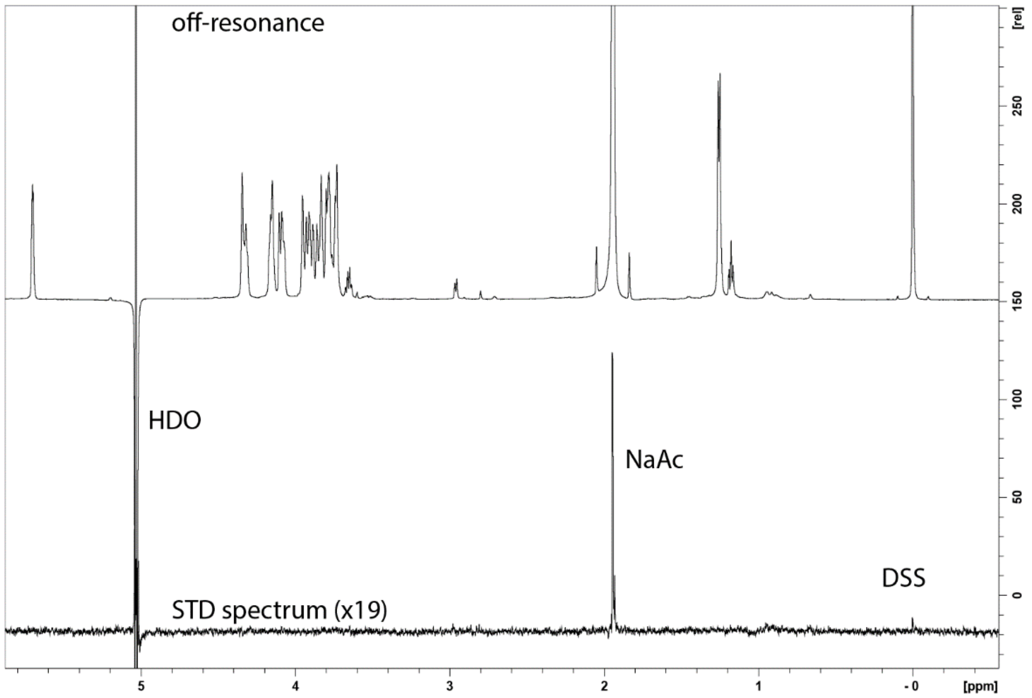

Fig S8 [continued]

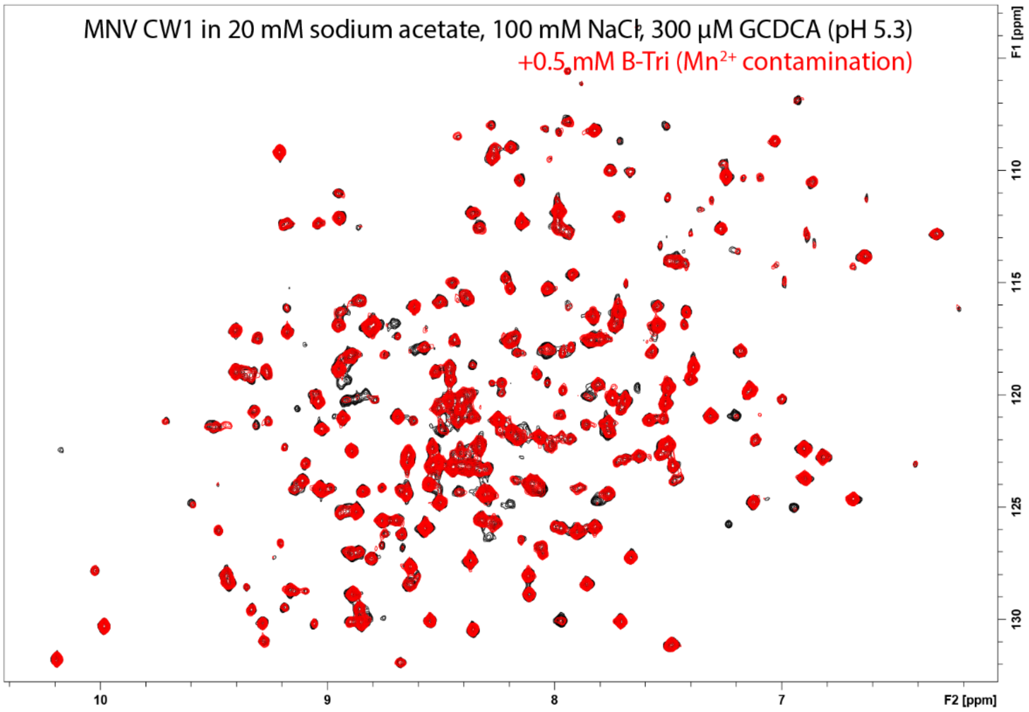

Fig S8 [continued]

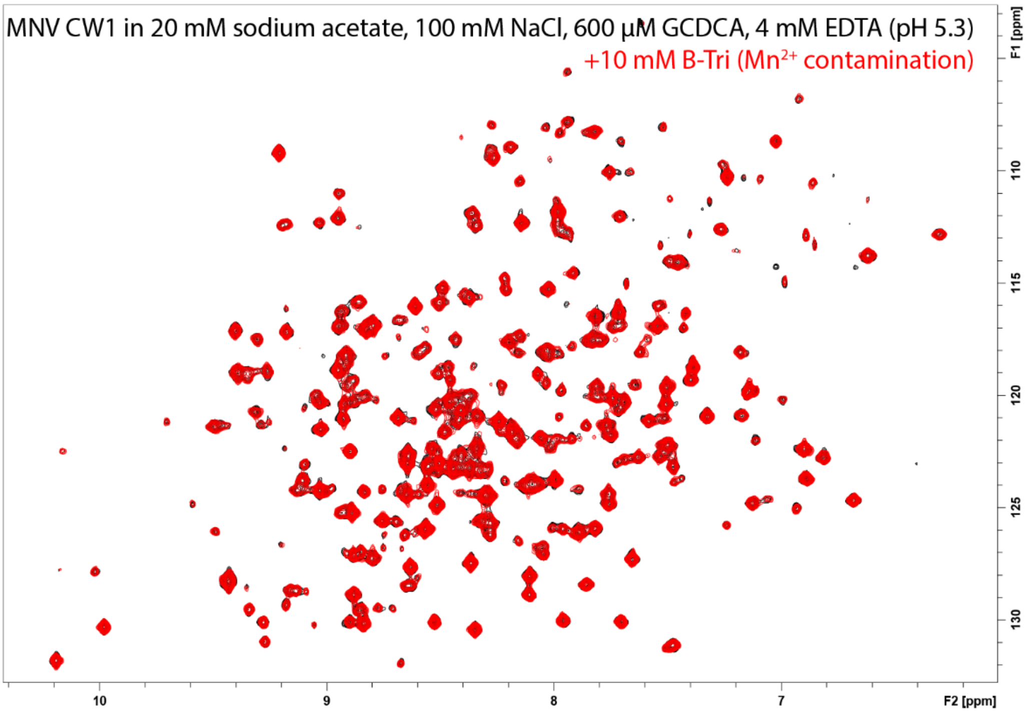

Fig S8 [continued]

MNV CW1 in 20 mM sodium acetate-d_3, 100 mM NaCl (pH 5.3), D_2O, 300 µM GCDCA + 4 mM GalNAc

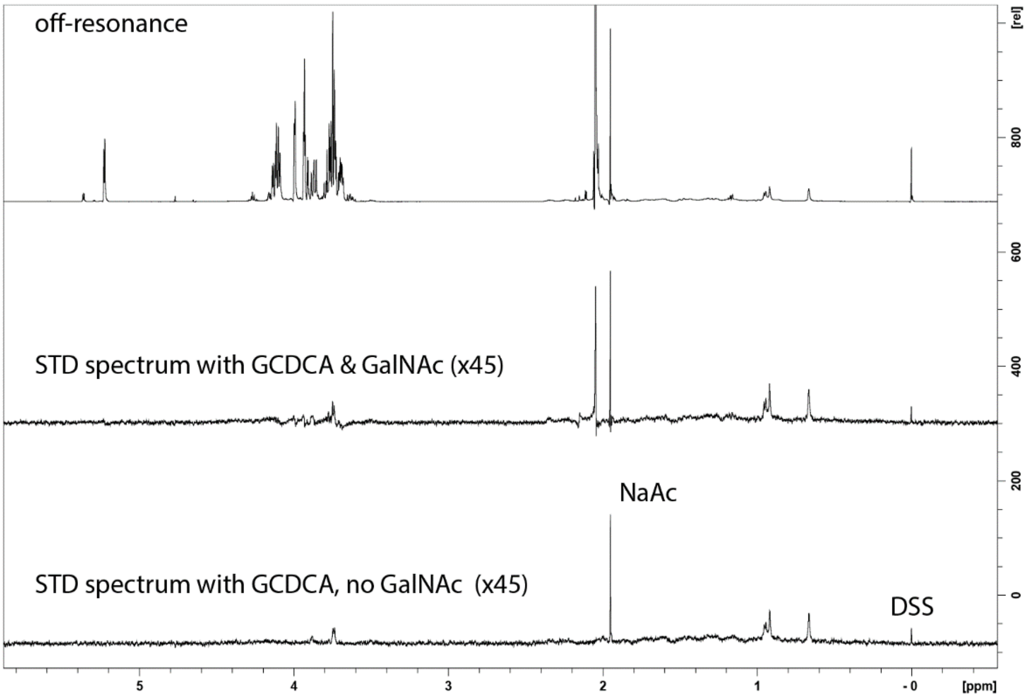

Fig S8 [continued]

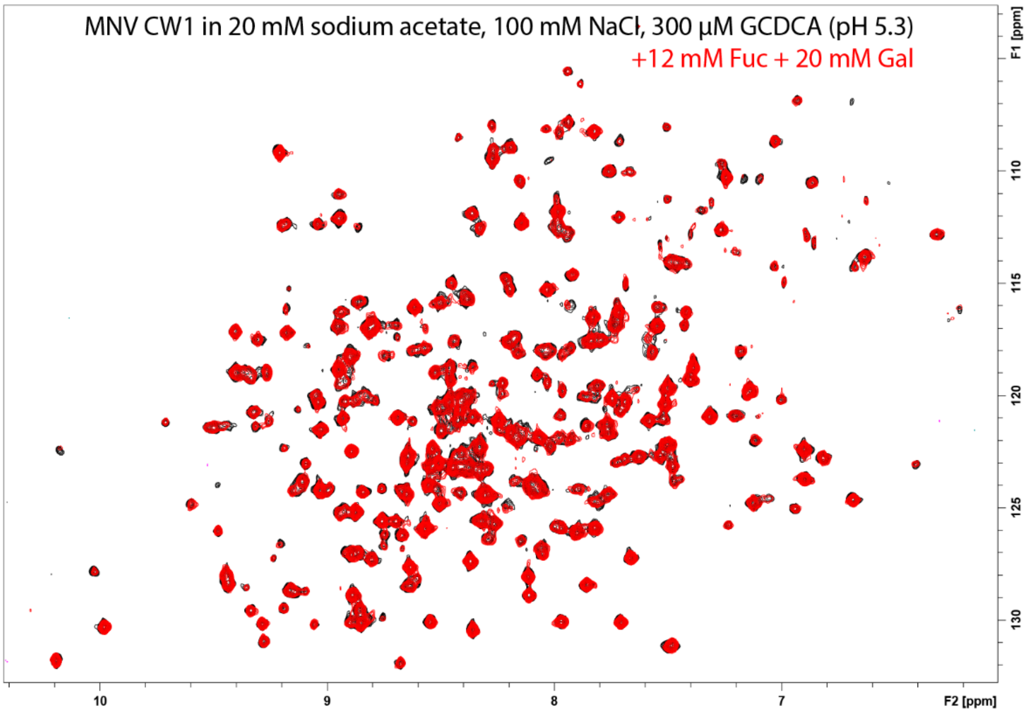

Fig S8 [continued]

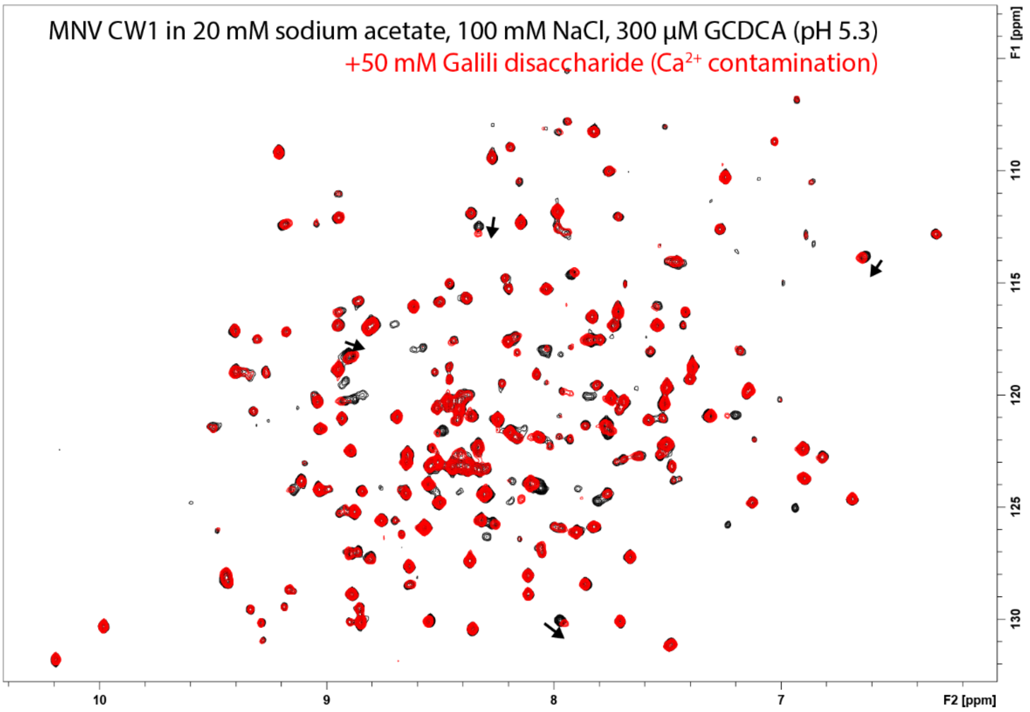

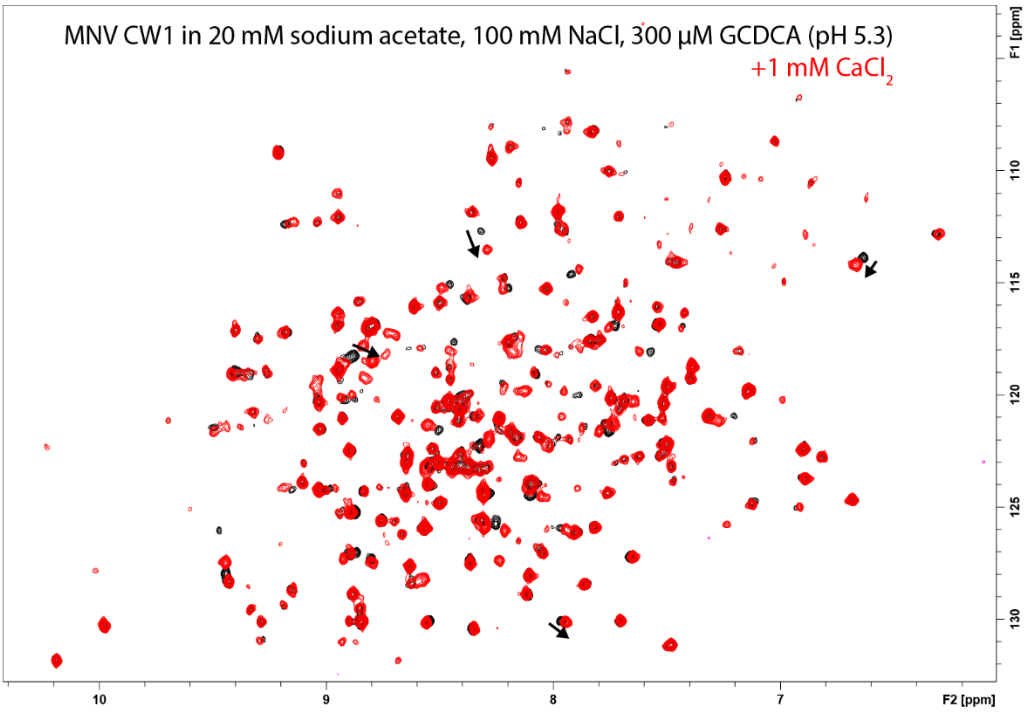

Fig S8 [continued]

MNV CW1 in 20 mM sodium acetate-d_3, 100 mM NaCl (pH 5.3), D_2O, 300 µM GCDCA + 2 mM Forssman antigen

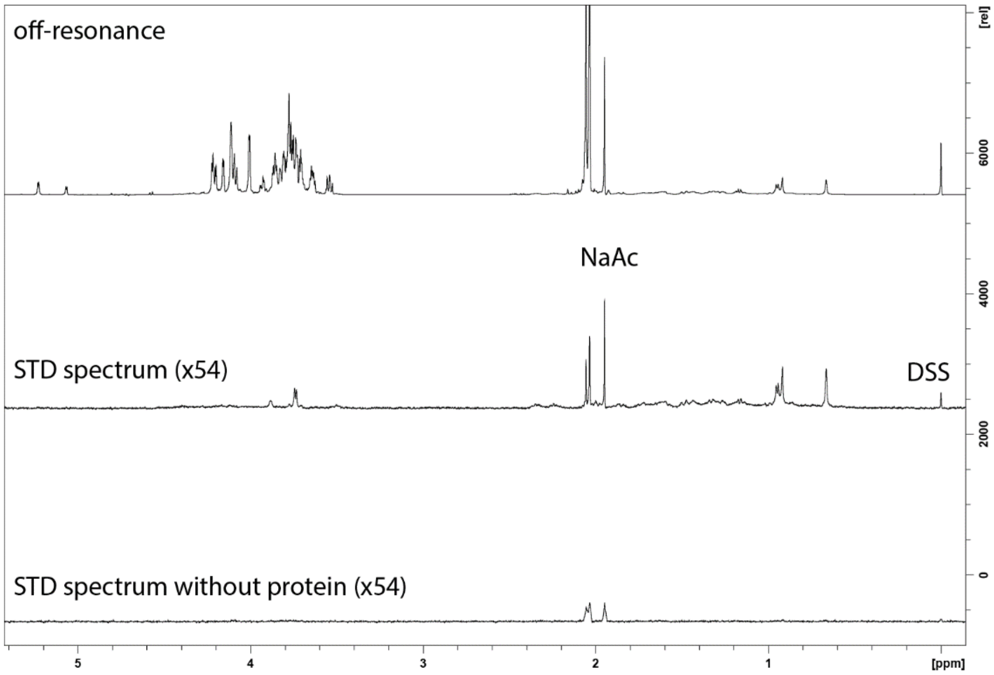

Fig S8 [continued]

MNV07 in 20 mM sodium phosphate, 100 mM NaCl (pH 6) + 2 mM Neu5Ac

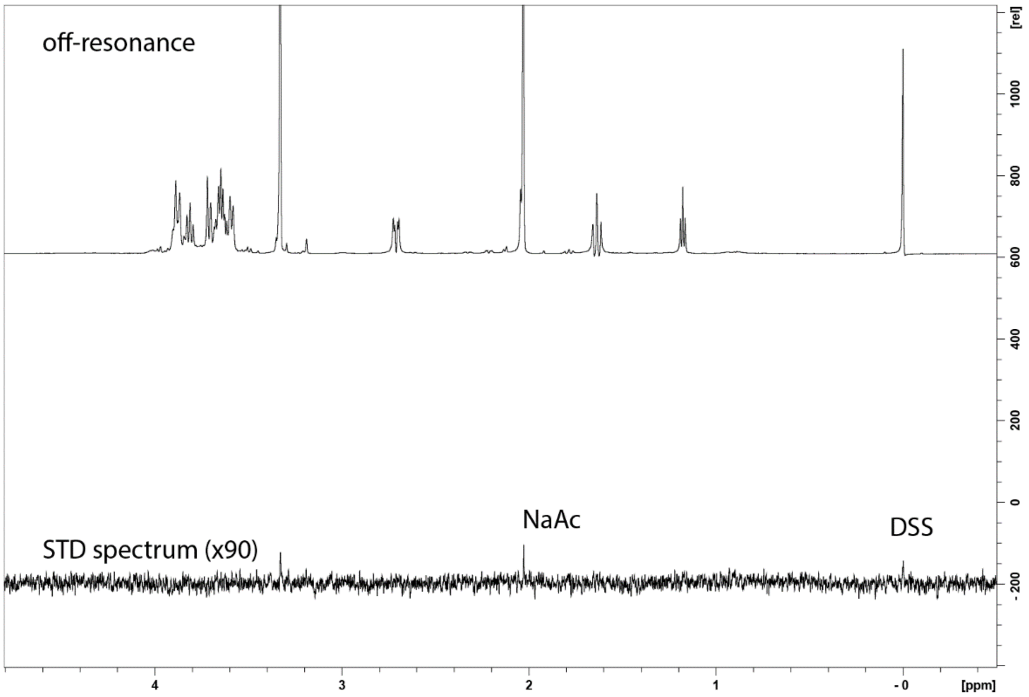

Fig S8 [continued]

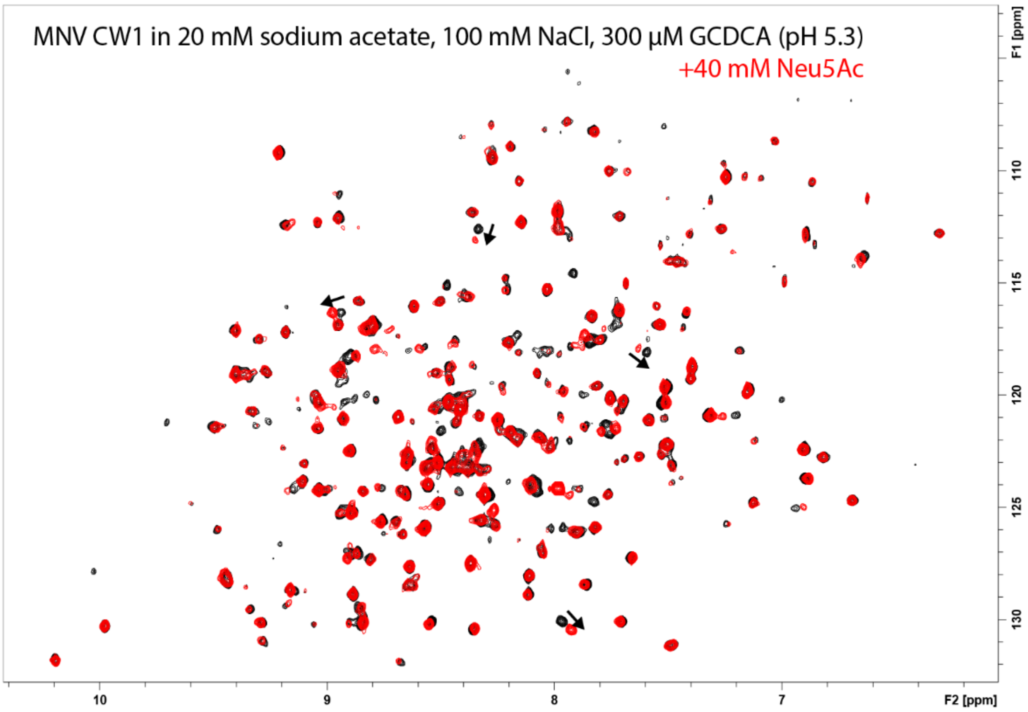

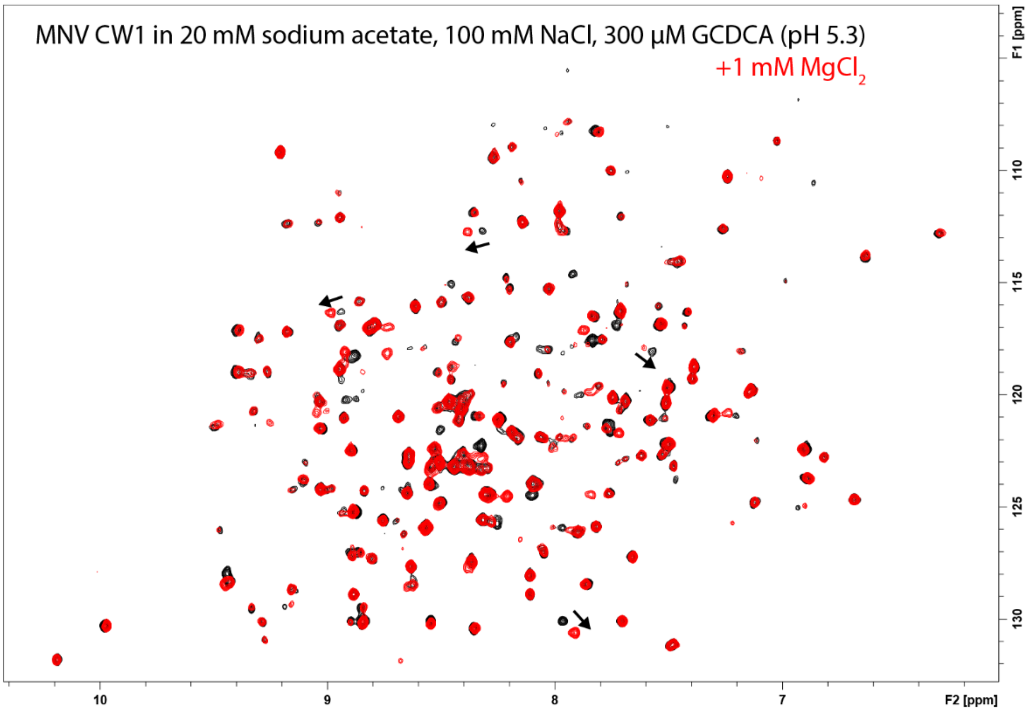

Fig S8 [continued]

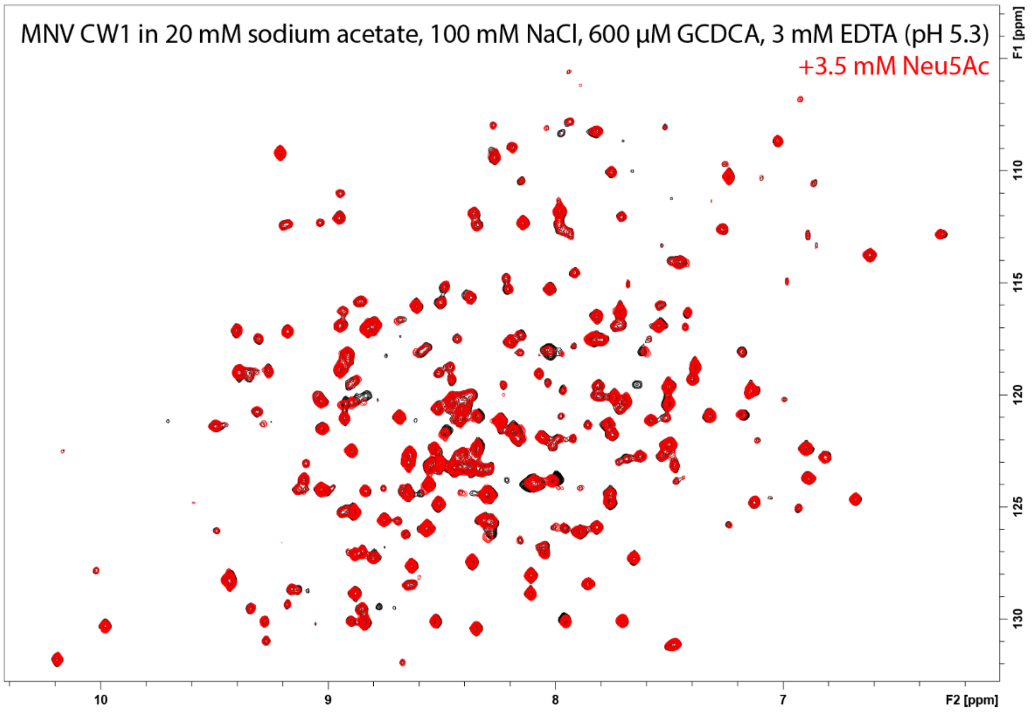

Fig S8 [continued]

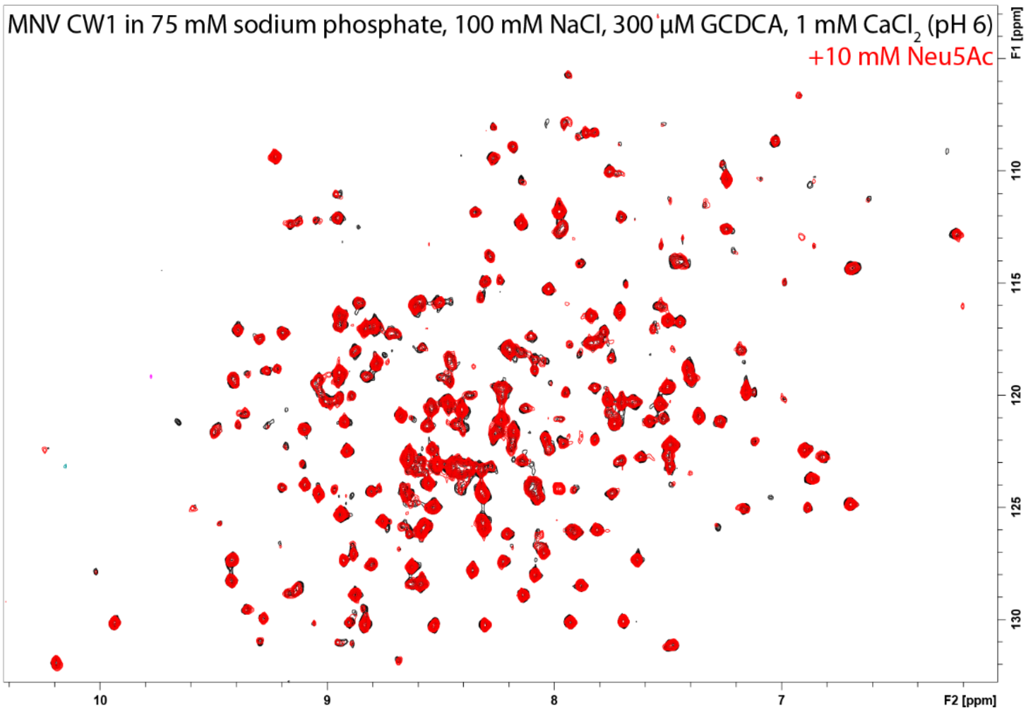

Fig S8 [continued]

MNV07 in 20 mM sodium acetate, 100 mM NaCl (pH 5.3),250 µM GCDCA + 2 mM 3‘SL

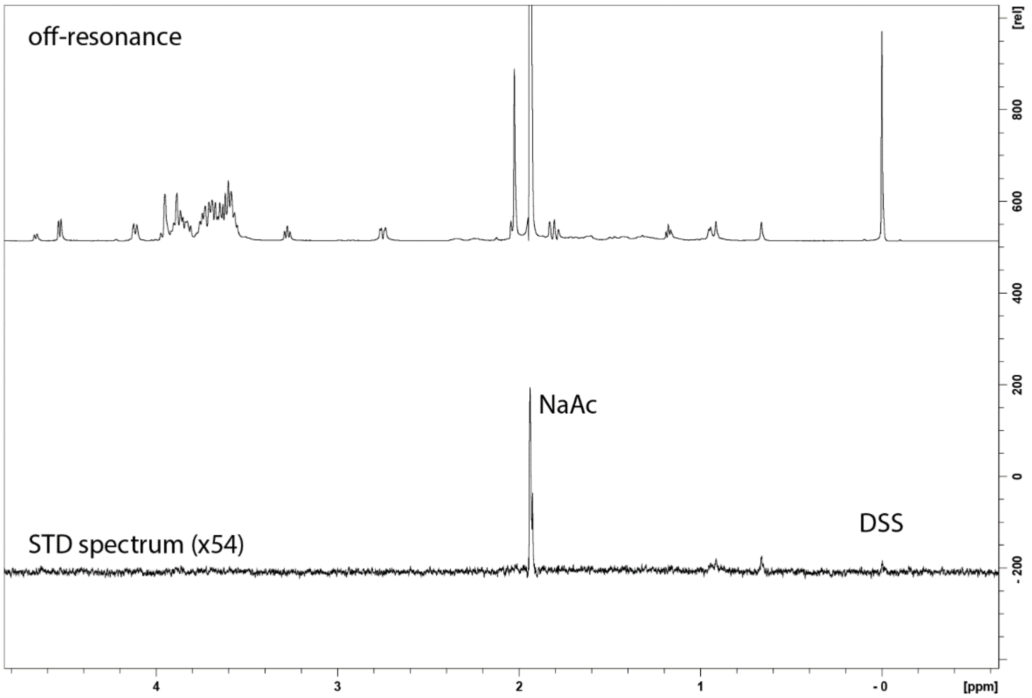

Fig S8 [continued]

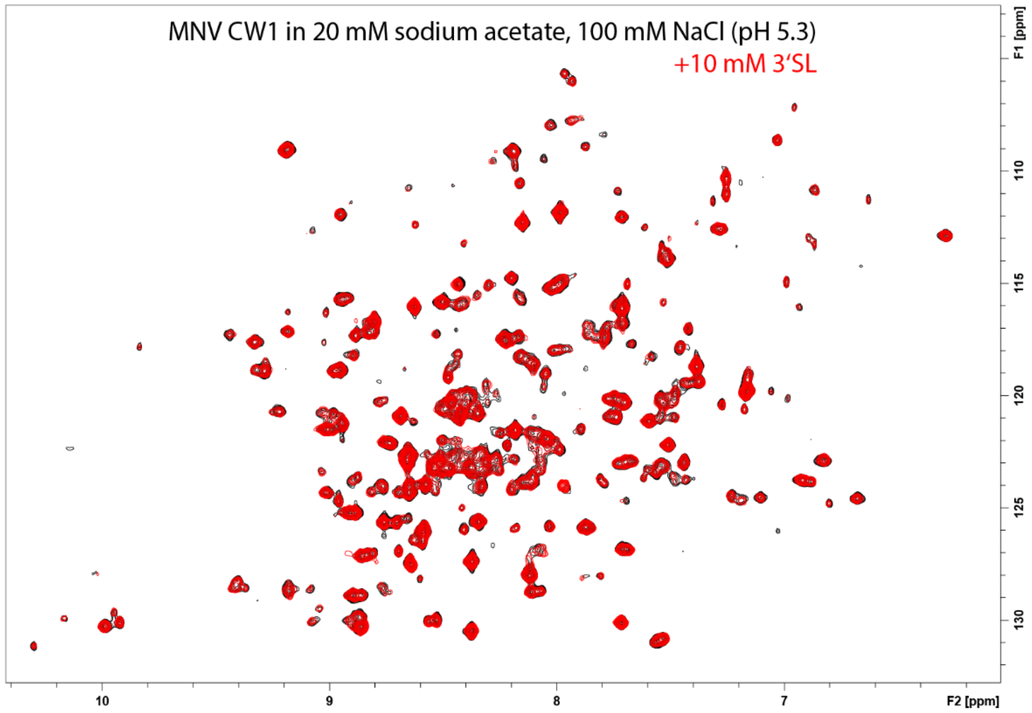

Fig S8 [continued]

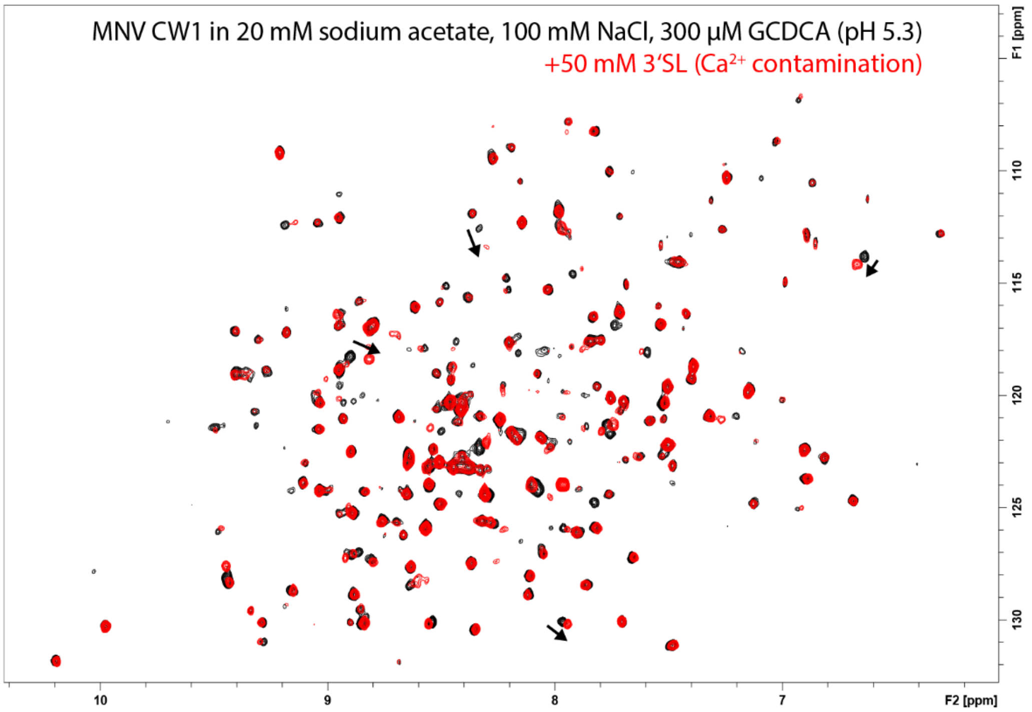

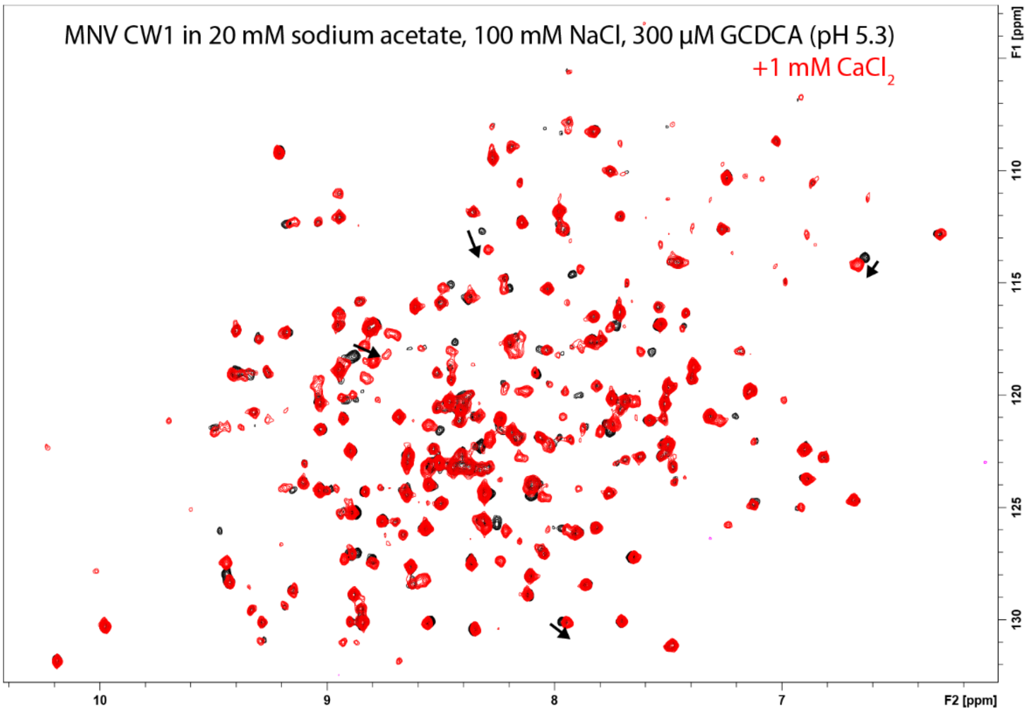

Fig S8 [continued]

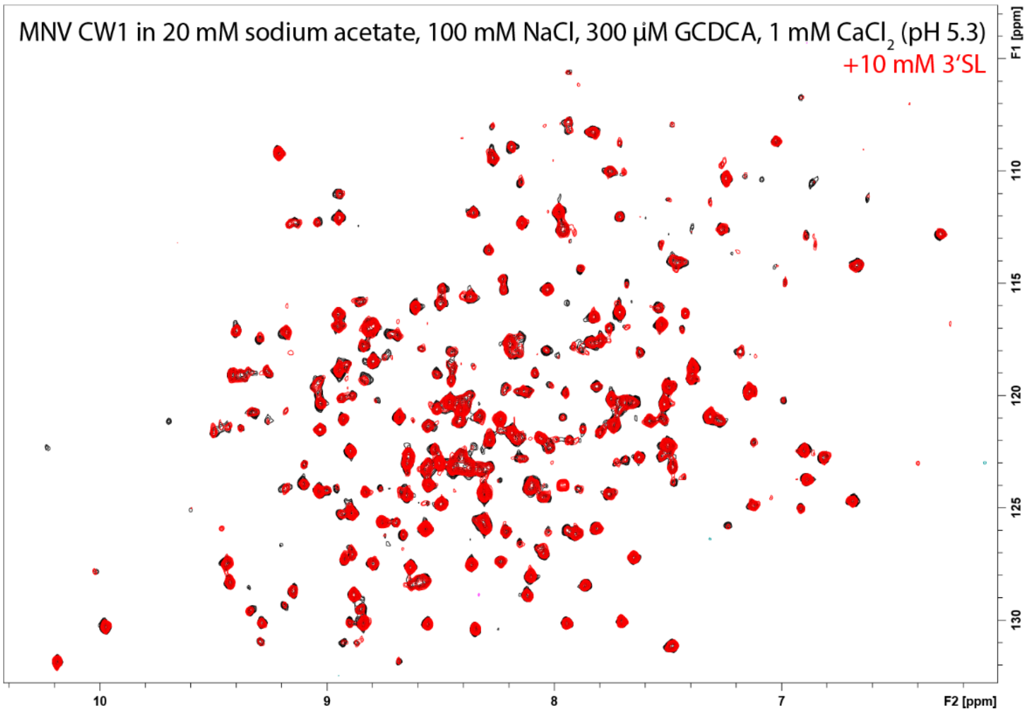

Fig S8 [continued]

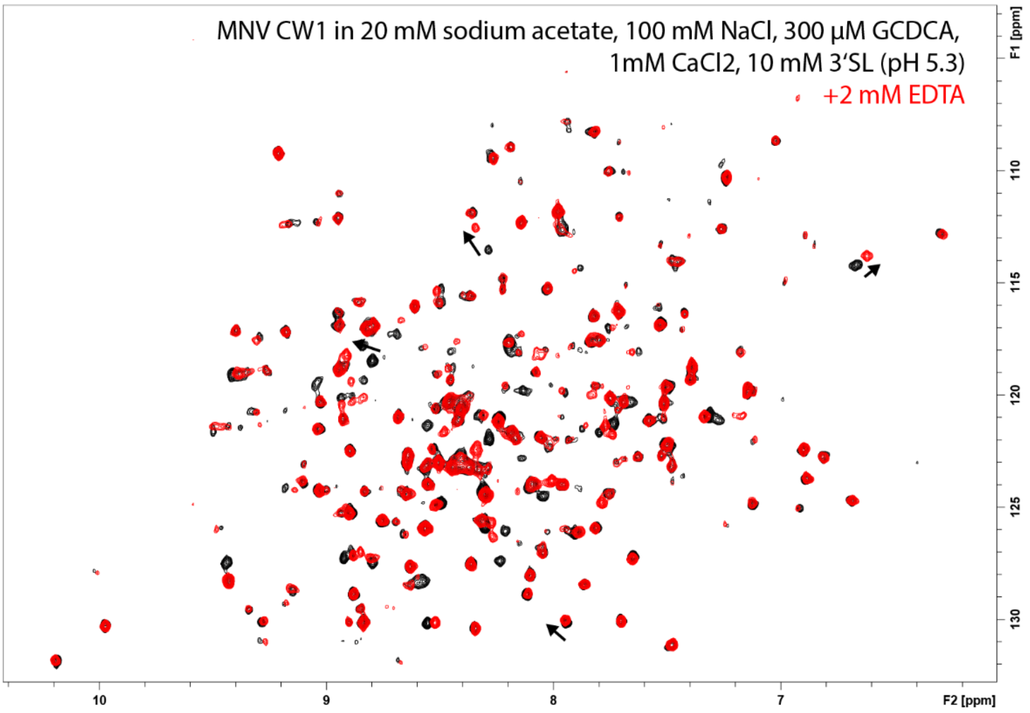

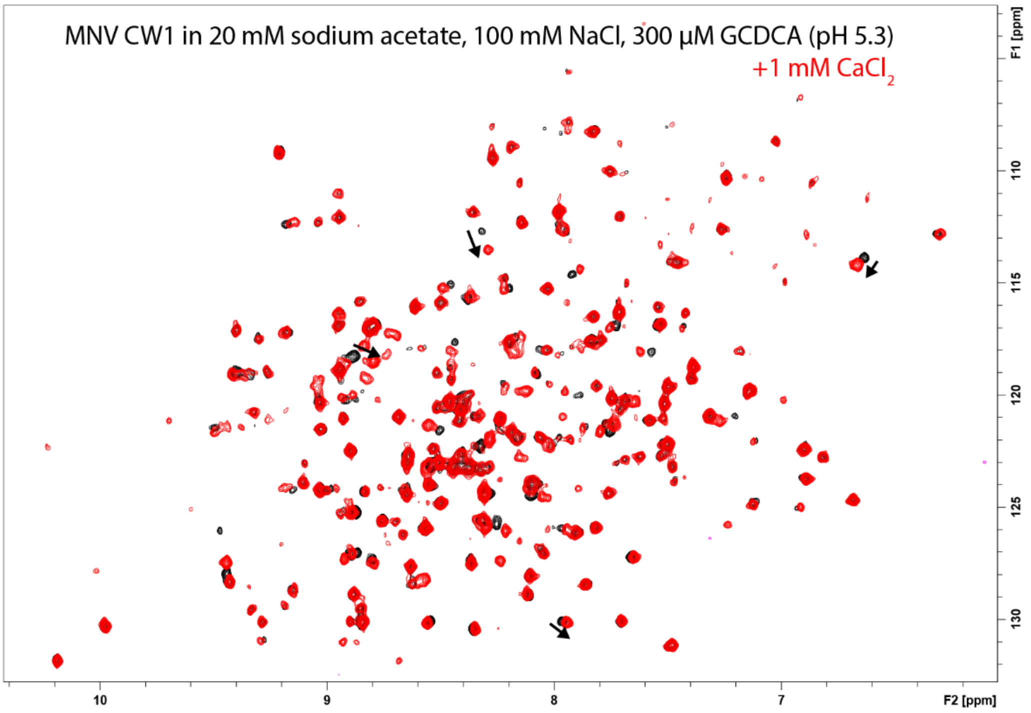

Fig S8 [continued]

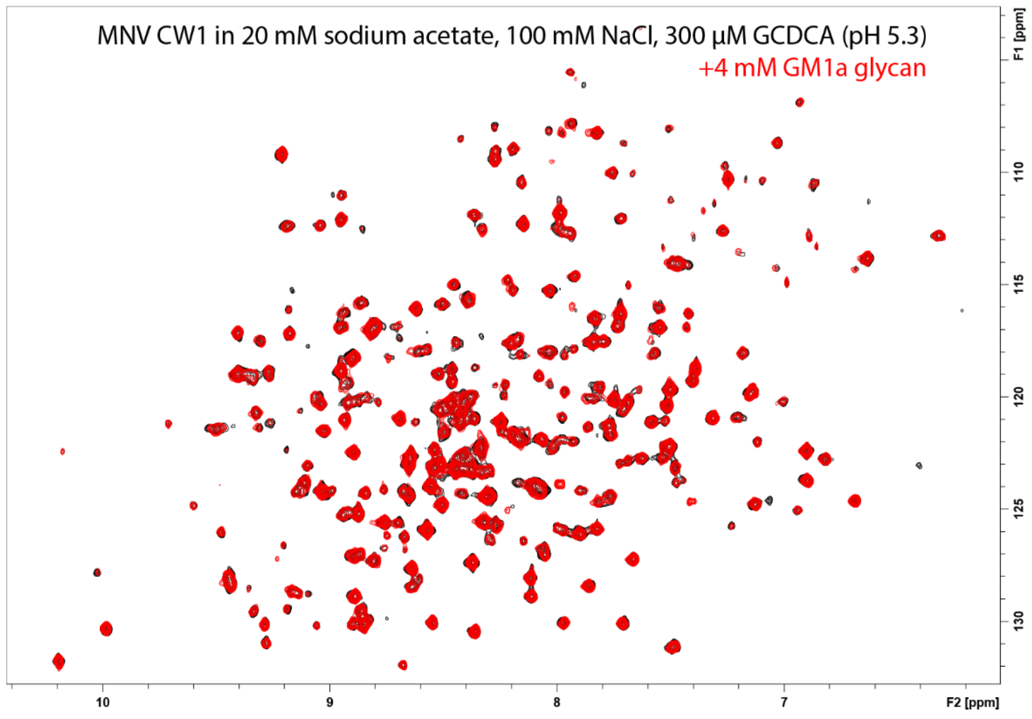

Fig S8 [continued]

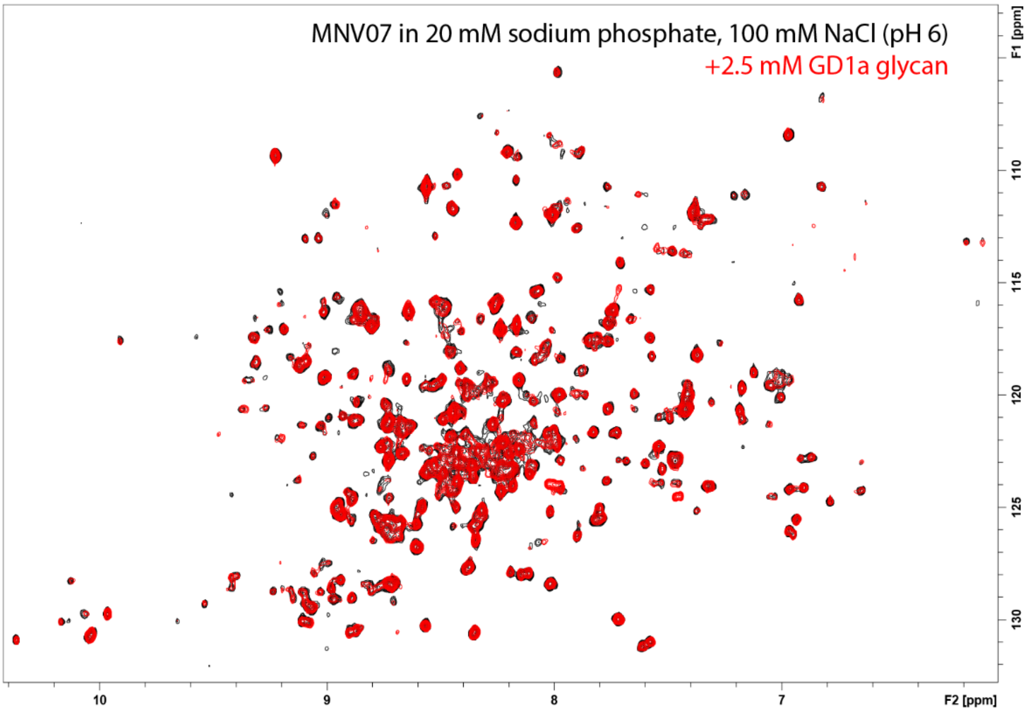

Fig S8 [continued]

Table S1. Experimental conditions for data in Table 2.

NoV strain (concentration)	Carbohydrate (concentration)	Buffer	Experiment (temperature)
MNV07 (24 µM)	**5** (2 mM)	20 mM sodium phosphate, 100 mM NaCl, pH 6	STD (277 K)
CW1 (200 µM)	**6** (10 mM)	20 mM sodium acetate, 100 mM NaCl, pH 5.3	CSP (298 K)
MNV07 (28 µM)	**7** (2.5 mM)	20 mM sodium phosphate, 100 mM NaCl, pH 6	CSP (293 K)
CW1 (40 µM)	**8** (4 mM)	20 mM sodium acetate, 100 mM NaCl, pH 5.3	STD (298 K)
MNV07 (41 µM)	**3** (5 mM)	20 mM sodium acetate, 100 mM NaCl, pH 5.3	CSP (298 K)
CW1 (200 µM)	**3** (10 mM)	20 mM sodium acetate, 100 mM NaCl, pH 5.3	CSP (298 K)
CW1 (72 µM)	**5** (40 mM)	20 mM sodium acetate, 100 mM NaCl, pH 5.3, 300 µM GCDCA	CSP (298 K)
MNV07 (80 µM)	**6** (2 mM)	20 mM sodium acetate, 100 mM NaCl, pH 5.3, 250 µM GCDCA	STD (277 & 298 K)
CW1 (72 µM)	**6** (50 mM)	20 mM sodium acetate, 100 mM NaCl, pH 5.3, 300 µM GCDCA	CSP (298 K)
CW1 (100 µM)	**14** (4 mM)	20 mM sodium acetate, 100 mM NaCl, pH 5.3, 300 µM GCDCA	CSP (298 K)
CW1 (50 µM)	**10** (4 mM)	20 mM sodium acetate-d_3, 100 mM NaCl, pD 5.3, 300 µM GCDCA (D_2O)	STD (298 K)
CW1 (100 µM)	**8** (12 mM), **9** (20 mM)	20 mM sodium acetate, 100 mM NaCl, pH 5.3, 300 µM GCDCA	CSP (298 K)
CW1 (100 µM)	**11** (50 mM)	20 mM sodium acetate, 100 mM NaCl, pH 5.3, 300 µM GCDCA	CSP (298 K)
CW1 (100 µM)	**12** (50 mM)	20 mM sodium acetate, 100 mM NaCl, pH 5.3, 300 µM GCDCA	CSP (298 K)
MNV07 (73 µM)	**3** (1.8 mM)	20 mM sodium acetate, 100 mM NaCl, pH 5.3, 140 µM GCDCA	STD (277 K)
CW1 (100 µM)	**3** (0.5 mM)	20 mM sodium acetate, 100 mM NaCl, pH 5.3, 300 µM GCDCA	CSP (298 K)
CW1 (50 µM)	**2** (2 mM)	20 mM sodium acetate-d_3, 100 mM NaCl, pD 5.3, 300 µM GCDCA (D_2O)	STD (298 K)
CW1 (50 µM)	**13** (2 mM)	20 mM sodium acetated-d_3, 100 mM NaCl, pD 5.3, 300 µM GCDCA (D_2O)	STD (298 K)
CW1 (70 µM)	**5** (10 mM)	75 mM sodium phosphate, 100 mM NaCl, pH 6, 300 µM GCDCA, 1 mM $CaCl_2$	CSP (298 K)
CW1 (70 µM)	**6** (10 mM)	20 mM sodium acetate, 100 mM NaCl, pH 5.3, 300 µM GCDCA, 1 mM $CaCl_2$	CSP (298 K)
CW1 (190 µM)	**5** (3.5 mM)	20 mM sodium acetate, 100 mM NaCl, pH 5.3, 600 µM GCDCA, 3 mM EDTA	CSP (298 K)
CW1 (70 µM)	**6** (10 mM)	20 mM sodium acetate, 100 mM NaCl, pH 5.3, 300 µM GCDCA, 1 mM $CaCl_2$, 2 mM EDTA	CSP (298 K)
CW1 (190 µM)	**3** (10 mM)	20 mM sodium acetate, 100 mM NaCl, pH 5.3, 600 µM GCDCA, 4 mM EDTA	CSP (298 K)

Table S2. Source of glycans.

Compound (Fig. 1)	Trivial name	Source
**1**	H-disaccharide (azide)	Donated by Prof. Javier Pérez-Castells
**2**	A-trisaccharide	Carbosynth
**3**	B-trisaccharide	cf. Materials and Methods
**4**	B-tetrasaccharide	Elicityl
**5**	Neu5Ac	Carbosynth
**6**	3'-sialyllactose	Elicityl
**7**	GD1a glycan	Elicityl
**8**	Fuc	Carbosynth
**9**	Gal	Fluka
**10**	GalNAc	Fluka
**11**	Galili disaccharide	Dextra
**12**	H-disaccharide (methyl glycoside)	in-house synthesis
**13**	Forssman antigen	Elicityl
**14**	GM1a glycan	Elicityl

Table S3: Assignment of blood group B trisaccharide 3.
Coupling constants have been evaluated as first order, and chemical shifts are referenced to DSS-d_6 (0.00 ppm).

Pyranose unit	Proton	Chemical shift (ppm)	$^{3}J_{HH}$ Coupling constants (Hz)		Carbon	Chemical shift (ppm)	$^{1}J_{CH}$ coupling constants (Hz)
**α-Gal-N_3 (Gal2)**	H1	5.69	H1-H2	4.4	C1	91.02	172.1
	H2	4.16	H2-H3	10.3	C2	75.07	147.8
	H3	4.08	H3-H4	3.1	C3	74.33	147.3
	H4	4.33	H4-H5	1.1	C4	67.03	146.3
	H5	4.07	H5-$H6_a$	7.8	C5	75.58	143.6
	-	-	H5-$H6_b$	4.8	-	-	-
	$H6_{a,b}$	3.78	-	-	C6	63.91	142.3
**α-Gal (Gal1)**	H1	5.20	H1-H2	3.9	C1	95.92	171.6
	H2	3.86	H2-H3	10.2	C2	70.88	147.0
	H3	3.92	H3-H4	3.3	C3	72.09	146.0
	H4	3.97	H4-H5	1.5	C4	72.11	146.2
	H5	4.31	H5-$H6_a$	6.0	C5	73.65	144.3
	-	-	H5-$H6_b$	6.0	-	-	-
	$H6_{a,b}$	3.73	-	-	C6	64.05	142.5
**α-Fuc**	H1	5.13	H1-H2	4.1	C1	103.24	173.8
	H2	3.80	H2-H3	10.1	C2	70.50	145.7
	H3	3.88	H3-H4	3.3	C3	72.06	144.8
	H4	3.83	H4-H5	1.6	C4	74.47	144.9
	H5	4.15	H5-CH_3	6.6	C5	70.33	141.6
	CH_3	1.26	-	-	C6	18.19	124.4

Table S4: NMR experiments and experimental conditions used for the assignment of blood group B trisaccharide 3.

The sample used for the assignment of α-azido B-trisaccharide **3** contained 50 mM of **3**, 75 mM sodium phosphate buffer at pH* 7.30, 100 mM NaCl and 100 µM DSS-d_6 in >99.95% D_2O. All experiments were acquired on a Bruker Avance III HD 600 MHz NMR spectrometer equipped with a TCI cryogenic probe at 298K.

N°	Experiment	Pulse program[1]	Number scans	Direct dimension (t_2)				Indirect dimension (t_1)				Relaxation delay (s)	Experimental time
				Offset (ppm)	Spectral width (ppm)	Data points	Acquisition time (ms)	Offset (ppm)	Spectral width (ppm)	Increments	Acquisition time (ms)		
1	^{1}H	zg, zgesgp	16	4.74	10	32768	2726, 2343	-	-	-	-	6	4 min
2	^{13}C	udeft	7168	95	230	20198	350	-	-	-	-	3.5	7 h
3	$^{1}H,^{1}H$ COSY	cosygpmfphpp	8	4.74	10	2048	170	4.74	12	512	35.5	1.7	2 h
4	$^{1}H,^{1}H$ TOCSY[2]	mlevphpp	8	4.74	10	2048	170	4.74	10	512	42.6	1.5	2 h
5	$^{1}H,^{13}C$ HMBC	hmbcgplpndqf	8	4.74	15	2048	136	60	140	512	29.1	2	2 h 22 min
6	$^{1}H,^{13}C$ H2BC (HMQC-COSY)	h2bcetgpl3	28	4.74	15	2048	136	60	100	128	3.6	2	2 h
7	$^{1}H,^{1}H$ NOESY[3]	noesygpphpp	16	4.74	10	2048	170	4.74	10	512	42.6	2.3	7 h 39 min
8	Edited $^{1}H,^{13}C$ HSQC	hsqcedetgpsisp2.2	4	4.74	12.5	2048	136	80	160	512	10.6	2	1 h 9 min
9	Non-decoupled $^{1}H,^{13}C$ HSQC	Modified hsqcetgpsp.3	4	4.74	12.5	2048	136	80	160	512	10.6	2	1 h 9 min
10	$^{1}H,^{1}H$ J-resolved 2D correlation	jresqpprpf	4	4.74	8	2048	213	4.74	0.05	512	17067	2	6 h

[1]From the standard Bruker library. [2] The experiment was acquired with a TOCSY mixing time of 200 ms. [3] 1 s NOE mixing time.

Table S5. Reevaluation of dissociation constants K_D based on previously reported titrations.

Buffer compositions were taken from the corresponding publications [2, 3] and are included here to assist comparison. Experiments with P-domains and VLPs were acquired at 298 K and 277 K, respectively.

N°.	NoV strain (protein)	Ligand	Buffer	K_D in mM	Ref
1	GII.4 Saga (P-domain)	Methyl α-L-fucopyranoside (**8**)	25 mM PBS pH* 7.40, 300 mM NaCl	15.6 ± 1.9	[2]
2	GII.4 Saga (P-domain)	Methyl α-L-fucopyranoside (**8**)	25 mM PBS pH* 6.50, 300 mM NaCl	18.4 ± 2.6	[2]
3	GII.4 Saga (P-domain)	Methyl α-L-fucopyranoside (**8**)	25 mM PBS pH* 5.50, 300 mM NaCl	21.9 ± 2.6	[2]
4	GII.4 Saga (P-domain)	Methyl α-L-fucopyranoside (**8**)	25 mM BisTris-d19 pH* 6.5, 300 mM NaCl	15.1 ± 1.6	[2]
5	GII.4 Saga (P-domain)	Methyl α-L-fucopyranoside (**8**)	25 mM sodium acetate-d3, pH* 4.50, 300 mM NaCl	24.5 ± 4.1	[2]
6	GII.4 Saga (P-domain)	Citrate	25 mM Tris-d11 pH* 7.40, 300 mM NaCl	22.9 ± 2.7	[3]
7	GII.4 Saga (P-domain)	H-disaccharide (**12**)	25 mM Tris-d11 pH* 7.40, 300 mM NaCl	26.5 ± 2.7	[2]
8	GII.4 Saga (P-domain)	β -Methyl glycoside of B-trisaccharide	25 mM Tris-d11 pH* 7.40, 300 mM NaCl	5.2 ± 0.5	[2]
9	GII.4 Ast6139 (VLPs)	β -Methyl glycoside of B-trisaccharide	25 mM Tris-d11 pH* 7.40, 300 mM NaCl	3.6 ± 0.2	[3]

References:

1. Okonechnikov, K.; Golosova, O.; Fursov, M.; team, U., Unipro UGENE: a unified bioinformatics toolkit. *Bioinformatics* **2012,** 28, (8), 1166-7.
2. Mallagaray, A.; Lockhauserbäumer, J.; Hansman, G. S.; Uetrecht, C.; Peters, T., Attachment of Norovirus to Histo Blood Group Antigens: A Cooperative Multistep Process. *Angew Chem Int Ed* **2015,** 54, (41), 12014-12019.
3. Mallagaray, A.; Rademacher, C.; Parra, F.; Hansman, G.; Peters, T., STD NMR Titrations Reveal Complex Multistep-Binding of L-Fucose to Norovirus Particles. *Glycobiology* **2017,** 27, (1), 80-86.
